# Developmental and tissue expression breadth define distinct but overlapping classes of housekeeping genes

**DOI:** 10.1371/journal.pcbi.1014787

**Published:** 2026-09-21

**Authors:** Alicia Lou, Juan F. Poyatos, Monica Chagoyen

**Affiliations:** 1 Centro Nacional de Biotecnologia (CNB-CSIC), Madrid, Spain; 2 Museo Nacional de Ciencias Naturales (MNCN-CSIC), Madrid, Spain; 3 Centro de Neurociencias Cajal (CNC-CSIC), Madrid, Spain; University of Notre Dame, UNITED STATES OF AMERICA

## Abstract

Housekeeping genes are commonly defined as broadly expressed genes that support essential cellular function. However, their identification in genome-wide datasets typically relies on spatial variation across adult tissues, while largely overlooking developmental time as an additional dimension of gene expression diversity. Here, we systematically compare gene expression breadth across developmental stages and adult tissues using zebrafish (*Danio rerio*) transcriptomic datasets that span embryogenesis and differentiated tissues. We classify genes according to their temporal expression breadth during development and examine how these patterns relate to traditional tissue-based definitions of housekeeping genes. As expected, developmental and adult expression breadth show only partial concordance: while a core set of genes is broadly expressed across both dimensions, many genes that are ubiquitous across adult tissues exhibit restricted expression during embryogenesis, and some genes expressed throughout development later become tissue-specific. Genes expressed across all developmental stages are enriched for fundamental cellular processes, show strong evolutionary conservation, and are associated with highly pleiotropic phenotypes. Notably, genes expressed during restricted developmental windows—particularly around gastrulation—can also play essential roles. These results highlight that expression ubiquity depends on both temporal and spatial context and underscore the importance of considering both dimensions explicitly when identifying and interpreting broadly expressed genes. More broadly, our analysis provides a framework for comparing developmental and tissue expression breadth and for exploring how gene expression dynamics across the life cycle relate to functional and evolutionary constraints.

## Author summary

Genes that are active in many conditions are called housekeeping genes. These genes are usually identified from adult tissues, because such data are the most widely available. However, this overlooks how gene activity changes during early development, leaving an incomplete picture of what it means for a gene to be broadly expressed. In this study, using zebrafish as a model, we compare how widely genes are expressed over time during embryonic development and across adult organs. These two patterns only partly overlap. Some genes are active throughout development, while others are widely expressed in adult tissues but only active during specific developmental stages, or vice versa. We show that genes active across all stages tend to carry out essential cellular roles, are highly conserved across species, and have widespread effects when disrupted. In contrast, genes with more limited activity are often involved in controlling specific developmental processes. Our findings demonstrate that developmental and adult tissue expression capture complementary aspects of gene ubiquity. This broader view helps clarify gene function across the life cycle.

## Introduction

Housekeeping (HK) genes are generally defined as genes that support fundamental cellular processes and are broadly expressed across biological contexts. In multicellular organisms, they are typically contrasted with cell- or tissue-specific genes, whose regulated expression underlies cellular identity and functional specialization. However, in practice, the term “housekeeping” is often used to encompass several related but distinct properties, including expression breadth, expression level, and stability across conditions. Here, we adopt a definition centered on expression breadth as the primary criterion, while considering expression level and stability as secondary, non-defining attributes that may further characterize subsets of broadly expressed genes.

Yet, expression breadth has been largely examined along a single axis: spatial variation across adult tissues. Most genome-wide efforts to identify HK genes therefore rely on transcriptomic technologies such as SAGE [1], microarrays [2–5], expressed sequence tags [6], and bulk RNA sequencing [7–10]. These studies have generated widely used HK gene catalogs in human and model organisms, but they primarily measure expression across differentiated tissues in mature organisms. A few comparative analyses across multicellular species have partially broadened this view [11], but they still rely predominantly on post-developmental tissues. As a result, HK definitions derived from these approaches implicitly treat gene expression ubiquity as a purely spatial property, without explicitly considering change across developmental time.

However, development represents an additional, largely orthogonal axis of gene expression variation. During embryogenesis, coordinated transcriptional programs drive cell fate specification, morphogenesis, and organ formation. Genes that appear ubiquitously expressed across adult tissues may nonetheless exhibit restricted temporal expression during development, whereas genes consistently expressed throughout embryogenesis may later become specialized in particular tissues. Although a limited number of studies have jointly analyzed developmental and adult tissue transcriptomes to define HK genes [12–14], the relationship between temporal expression breadth during development and spatial expression breadth across adult tissues has not been systematically quantified.

Here, we address this problem by comparing temporal and spatial expression breadth. Using zebrafish (*Danio rerio*) as a model system, we classify genes according to their developmental expression breadth using a dense RNA-seq time course spanning embryogenesis and early larval stages [15]. We then directly compare these classifications with adult tissue expression breadth derived from a zebrafish tissue atlas [16]. This integrated approach allows us to quantify concordance and discordance between developmental and adult expression breadth and to identify gene classes that would not be detectable using either dimension alone.

Beyond classification, we systematically characterize these developmental expression-breadth classes across multiple biological dimensions, including gene ontology enrichment, transcription factor and cofactor representation, phenotype-based pleiotropy and lethality annotations, and evolutionary properties such as gene age, orthology, and paralogy. Finally, we interpret these patterns within the framework of evolutionary biology, examining how developmental expression breadth relates to the developmental hourglass model [17].

By explicitly comparing temporal and spatial transcriptomic data, our approach provides a computational perspective on gene expression ubiquity across the life cycle. More broadly, it illustrates how multidimensional expression breadth can help refine functional gene classifications and facilitate the exploration of links between transcriptomic patterns and developmental and evolutionary constraints.

## Results

### Ubiquitous and stage-specific gene classes during vertebrate development

**Fig 1:**
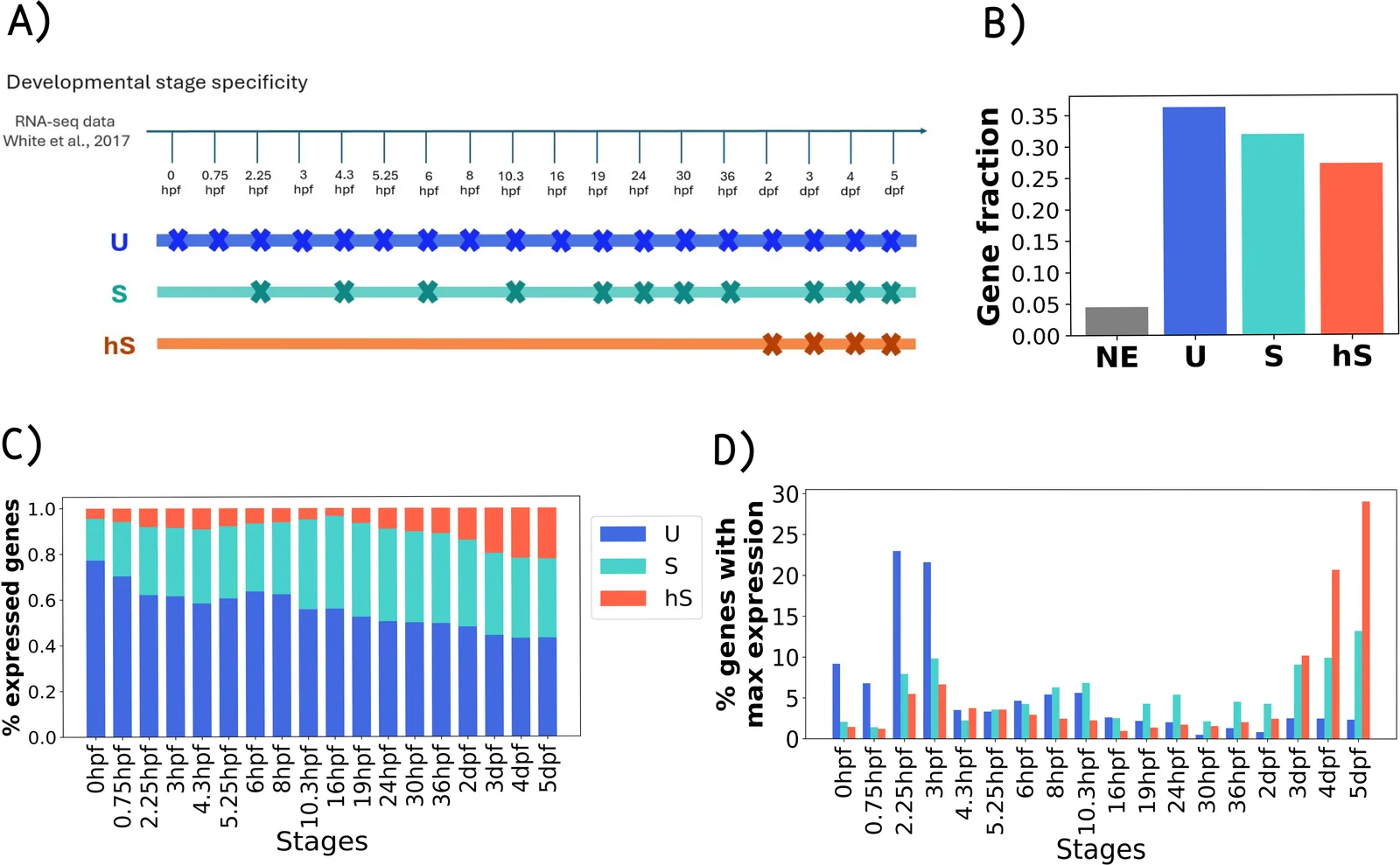
Gene classification based on developmental specificity. **A)** Developmental classification based on bulk RNA-seq embryos [15]. The arrow represents all the developmental stages identified in the bulk data. Below, we show one example gene for each class. The X’s indicate that the gene is expressed at that stage (TPM > 1). In all the panels, Ubiquitous (U) genes are represented in dark blue, specific (S) genes in light blue, and highly specific (hS) genes in red. **B)** Fraction of genes in each gene class. Non-Expressed (NE) genes (those with TPM < 1) are represented in gray. **C)** Proportion of genes expressed in each stage per gene class. **D)** Distribution of genes by their stage of maximum expression (per developmental class). Each bar represents the percentage of genes in a given class that reach their highest expression at a specific developmental stage. For each gene class, the cumulative percentage across all stages adds up to 100% (hpf, hours post fertilization).

We summarized the continuum of expression breadth during development into three gene categories based on an RNA-seq time-course dataset spanning 18 stages of zebrafish embryogenesis ([15]; Methods): *Ubiquitous* (U) genes (*n* = 8,579), expressed across all stages; *Specific* (S) genes (*n* = 7,551), expressed in ≥50% of stages; and *highly Specific* (hS) genes (*n* = 6,446), expressed in <50% of stages (S1 Table and Fig 1A). An additional 1,066 genes did not meet expression thresholds (TPM > 1) (Fig SAA and SAB in S1 Text). This partition divides genes into three equal groups (tertiles) based on the proportion of developmental stages in which they are expressed (Fig 1B), and largely recapitulates distinct groups defined by alternative continuous metrics of temporal specificity, τ [18] and temporal variability (coefficient of variation, CV) (Fig SAC and SAD in S1 Text; Methods). They are also consistent with the widely used conceptual distinction between broadly expressed (“housekeeping”) and context-specific genes, while accommodating intermediate patterns of expression to be represented. Applying the same classification procedure to an independent bulk RNA-seq developmental dataset [19] yielded highly concordant gene assignments, supporting the robustness of the U, S, and hS categories across independent transcriptomic datasets (Fig SAE in S1 Text).

As development progresses, the number of S and hS genes expressed increases, thus making the percentage of U genes expressed at each stage decline (Fig 1C). Despite this decline, U genes remain the largest and most stable fraction of the transcriptome, accounting for approximately 90% of total transcripts in early stages and about 71% in later stages (Fig SAF and SAG in S1 Text). U genes also display the lowest average expression variability across the five pooled biological replicates (12 embryos per replicate) during early embryogenesis (up to 16 hpf), after which their variability becomes comparable to that of S genes (Fig SAH in S1 Text). Interestingly, hS genes exhibit the lowest expression variability during 4 and 5 dpf, coinciding with the developmental stages at which this gene class becomes more broadly expressed.

It is also informative to examine maximum expression. Although this metric does not capture the full temporal dynamics of gene expression, it provides a simple summary of the developmental period in which a gene is most transcriptionally active. As a complement to the developmental breadth classification, the distribution of peak expression stages helps reveal whether genes from different classes tend to concentrate their highest expression at distinct phases of development (Fig 1D; Methods). U genes show their highest expression predominantly during zygotic genome activation (2.25-3 hpf), even though they account for most expression across early and mid-embryonic stages (Figs 1C and SAF in S1 Text). S genes exhibit a biphasic pattern, with peak expression occurring in both early and late stages. In contrast, and consistent with the progressive increase in overall transcription during development (Fig SAI in S1 Text), most hS genes reach their maximum expression at the latest developmental stages (Fig 1D). Thus, beyond the differences in expression breadth, the distinction between S and hS genes also mirrors key developmental transitions.

### Developmental gene classes differentiate function and regulation

**Fig 2:**
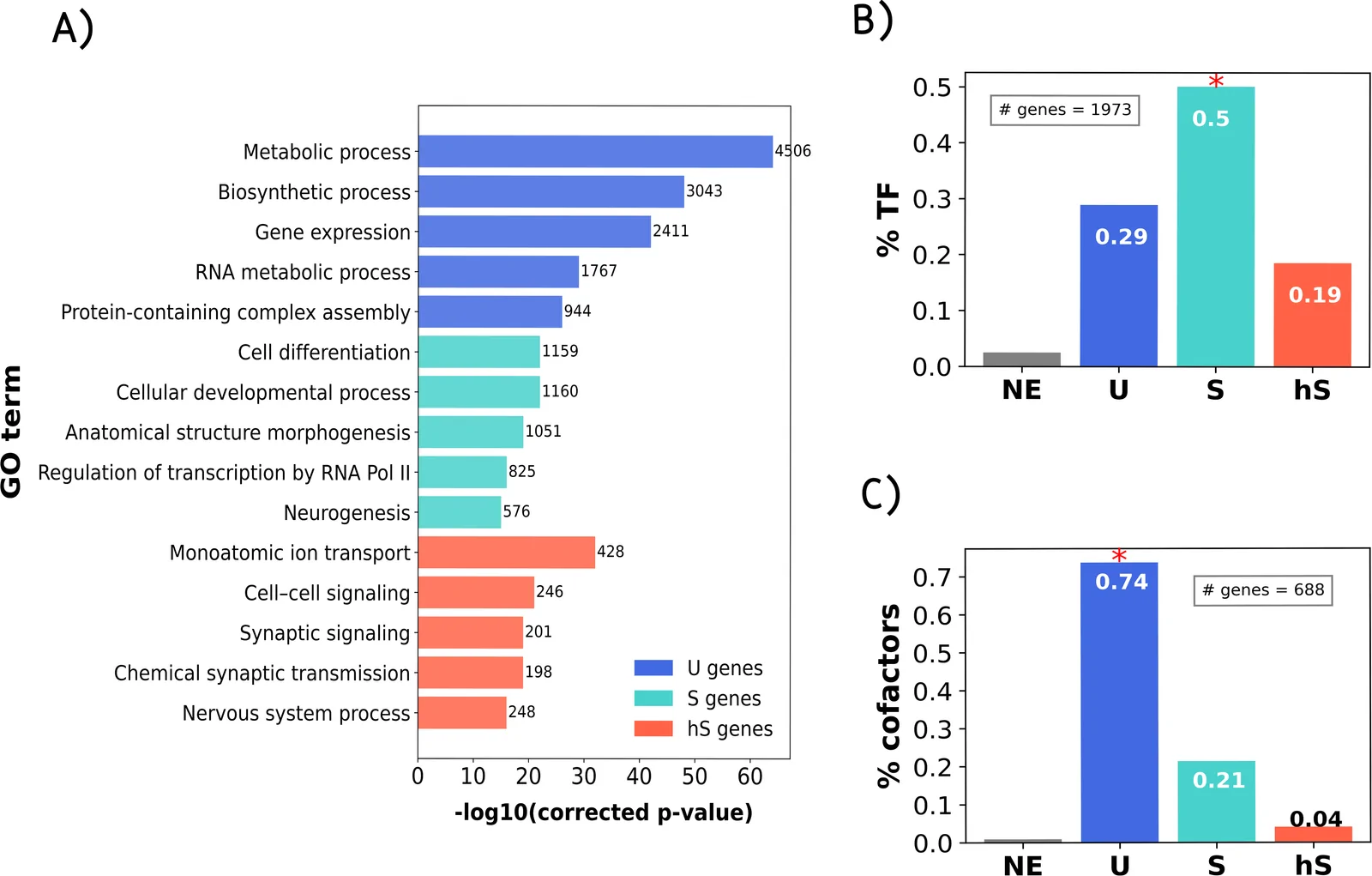
Developmental classes correspond to different functions and regulations. **A)** Enriched GO terms for each gene class: U (dark blue), S (light blue), hS (red). The y-axis lists GO terms selected from S2 Table. Numbers next to each bar indicate how many genes are associated with that term, and enrichment significance is shown by the corrected p-value on the x-axis. **B)** Fraction of transcription factors (TFs) in each class relative to the total TFs in the dataset. **C)** Fraction of cofactors in each class relative to the total cofactors in the dataset. Asterisks above bars in (B) and (C) indicate classes significantly enriched based on a hypergeometric test (Methods, p-value < 0.0001).

Nonetheless, it is reasonable to question how arbitrary this classification may be. To assess its biological validity, we first examined the functional associations of each group (Fig 2A and S2 Table; Methods). As expected, U genes are enriched in core cellular functions such as biosynthesis and metabolic processes, RNA related processes, gene expression, and protein complex organization. By contrast, the two specific classes capture more specialized roles. S genes are enriched in transcriptional regulation and developmental processes, including cell differentiation, morphogenesis, and neurogenesis. hS genes, in turn, are associated with cell–cell and nervous system processes such as signaling, ion transport, and synaptic activity. These processes typically emerge by ~20–30 hpf and continue developing into larval stages as neural circuitry matures.

We then compared transcription factors (TFs) and cofactors (including coactivators, corepressors, chromatin remodelers, mediator components, and TAFs), hypothesizing that their developmental expression patterns would reflect their distinct regulatory roles. Indeed, TFs remain largely inactive during the earliest stages and become expressed only after zygotic genome activation or during differentiation.

This pattern is consistent with their predominant classification as S genes (50%, n = 988), with limited association to U (29%, n = 570) or hS (19%, n = 366) categories (Fig 2B). Note that only the association with S genes remains statistically significant when testing for enrichment in each group (Methods, hypergeometric test, p-value = 1.48e-68). Earlier results in humans [20], mouse [21], and other metazoan systems [22] corroborate this result. By contrast, cofactors are more evenly required throughout development, with the majority (74%, *n* = 507; p-value = 2.88e-91) falling within the U class, and only smaller and non-significative fractions in the S (21%, *n* = 147) or hS (4%, *n* = 28) groups (Fig 2C).

### Partial concordance between developmental and adult tissue gene expression classes

**Fig 3:**
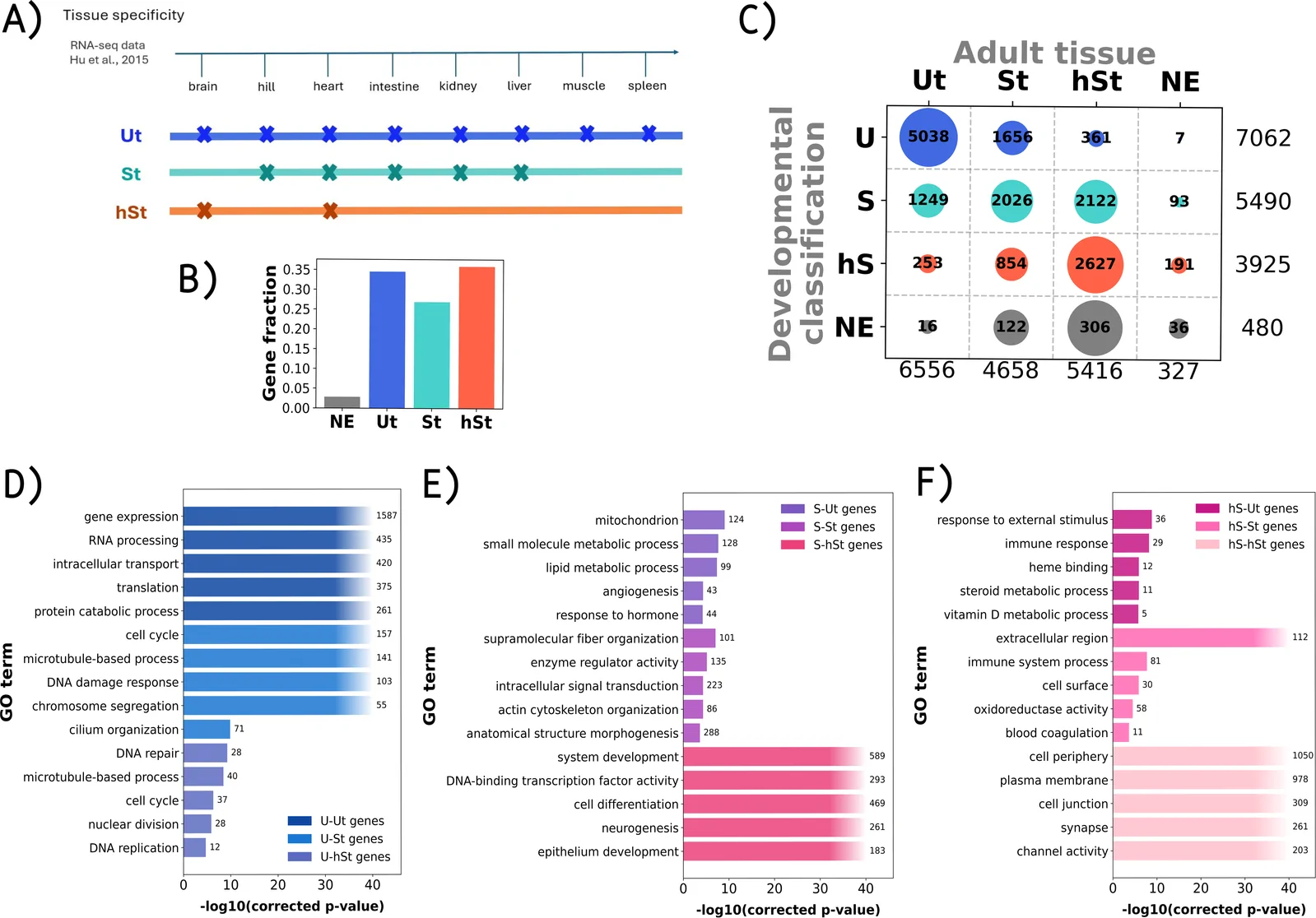
Gene classification based on tissue specificity. **A)** Adult tissue classification based on bulk RNA-seq data [16]. The blue arrow depicts the set of tissues analyzed. Below, we present one example gene from each class, with X’s indicating the tissues in which that gene is expressed (TPM > 1). **B)** Fraction of genes in each class. **C)** Comparison of developmental and tissue-based classifications. Points show the proportion of genes in each developmental class (U, S, hS) assigned to each tissue category (Ut, St, hSt), with point size indicating the row-wise percentage. While most genes retain their class, some fall outside the diagonal, indicating differences between the two schemes. **D–F)** Selected enriched Gene Ontology (GO) terms for **(D)** U genes subdivided into U-Ut, U-St, and U-hSt. **(E)** S genes subdivided into S-Ut, S-St, and S-hSt. **(F)** hS genes subdivided into hS-Ut, hS-St, and hS-hSt. For each enrichment analysis, the corresponding parent gene class was used as the statistical background (e.g., all U genes for U subclassifications). Numbers adjacent to each bar indicate the total number of genes annotated to each term. The x-axis shows enrichment significance as −log_10_(adjusted *p*-value). Bars with faded ends indicate *p*-values that underflow to zero because they exceed the lower limit of 64-bit floating-point representation; these correspond to *p*-values < 2.225 × 10^−308^.

If our developmental classification captures meaningful biological features, how does it compare to the *standard* definition of HK genes in adulthood? To address this question, we analyze bulk RNA-seq data from eight major adult zebrafish tissues –brain, gill, heart, intestine, kidney, liver, muscle, and spleen [16] (Methods, Fig 3A). Genes were categorized into three groups as before but this time based on adult tissue expression patterns (Methods, S1 Table): *ubiquitous tissue* (Ut) genes, *tissue-specific* (St) genes, and *highly tissue-specific* (hSt) genes (Figs 3B, SBA and SBD in S1 Text).

When compared with developmental expression profiles (Fig 3C and S1 Table), approximately 71% of U genes retained ubiquitous expression in adult tissues (Ut type), indicating only partial conservation and suggesting post-embryonic, context-dependent regulation. These (U,Ut) genes are strongly enriched for core cellular processes, including RNA processing and translation, hallmarks of traditional HK function (Fig 3D and S3 Table; Methods). In contrast, (U,St) and (U,hSt) classes were enriched for cell cycle regulation, DNA repair, and microtubule-based processes, consistent with their peak activity during early developmental stages characterized by high proliferative demand. To test whether U-St and U-hSt genes exhibit significantly lower expression in adult tissues compared to concordant specific (S-St) and highly specific (hS-hSt) baselines, we performed a one-sided Wilcoxon rank-sum test. We found that while U-St genes showed no significant reduction, U-hSt genes were significantly less expressed (p-value < 0.05) across multiple tissues (brain, intestine, kidney, liver, and spleen).

S genes generally retained specific or highly specific expression in adult tissues. Specifically, (S,St) were enriched for functions in supramolecular fiber organization, enzyme regulation, and intracellular signaling (Fig 3E and S3 Table). Moreover, (S,hSt) genes are associated with developmental processes, TF activity and cellular differentiation, including neurogenesis, peaking around 3 days post-fertilization (dpf) as brain structures mature. Intriguingly, a notable subset (~23%) of genes with stage-specific developmental expression displayed ubiquitous expression in adult tissues (S,Ut). These genes were predominantly associated with mitochondrial localization and small molecule metabolic processes, reflecting the transition toward increased metabolic demand in later embryonic stages [23,24].

Among hS genes, 67% remain highly specific in adult tissues (hSt). These genes are enriched for functions at the cell periphery, including plasma membrane localization, synaptic and cell-junction components, and ion channel activity (Fig 3F and S3 Table). In addition, hS genes classified as St show enrichment in extracellular matrix organization, cell surface signaling, and immune response; functions broadly used across adult tissues but typically activated later in development. Notably, a small subset of hS genes becomes ubiquitously expressed in adulthood (hS,Ut), with roles in immune regulation and vitamin D metabolism, suggesting functions that are dispensable in early development but essential across tissues later in life.

Finally, expression level also varies across subclasses, with hSt genes showing significantly lower average expression than other subcategories (Fig SBE in S1 Text).

### Distinct properties of developmental gene classes

These analyses demonstrate that our developmental classification adds a temporal dimension to the *standard* HK framework, revealing distinctions not apparent in adult tissues. This observation motivates further examination on its relationship to pleiotropy, gene family history, and evolutionary age.

#### Pleiotropy is not always coupled to developmental ubiquity of expression.

**Fig 4:**
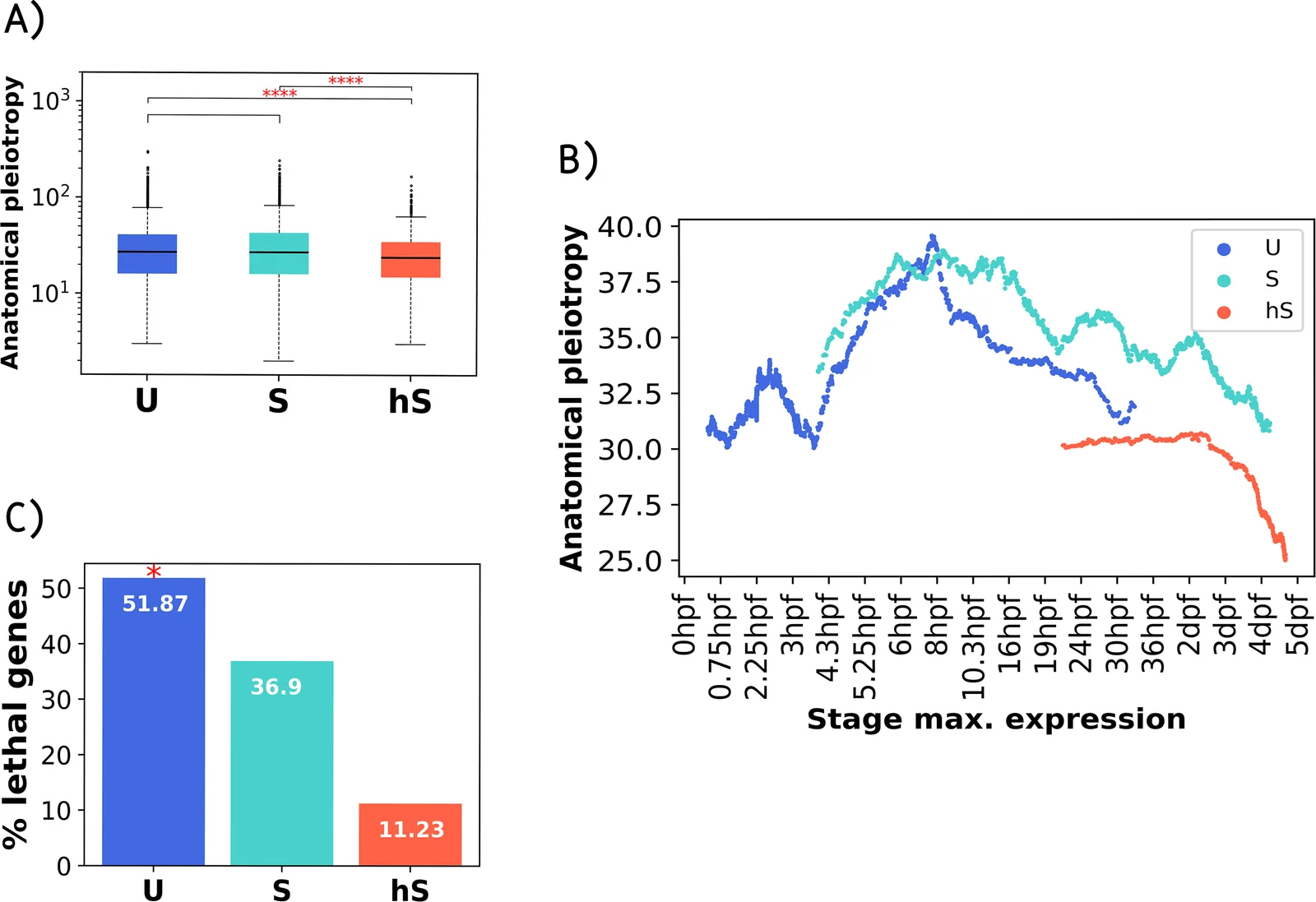
Anatomical pleiotropy analysis. **A)** Anatomical pleiotropy distribution among developmental classes. **B)** Average anatomical pleiotropy by stage of maximal expression, per gene class (sliding window = 400, note that ~26%, ~ 22% and ~12% of U, S, and hS genes, respectively have associated phenotypic effects). The apparent absence of S and hS genes at the earliest stages reflects missing anatomical annotations rather than absence of expression, as some reach maximal expression (Fig 1D) but likely lack associated phenotypic data. **C)** Fraction of genes with a ‘lethal’ phenotype in each class (U, S, hS), relative to all ‘lethal’ genes in the dataset. Asterisk indicates U class is significantly enriched based on a hypergeometric test (Methods, p-value < 0.0001).

For each gene, we quantified its organism-level phenotypic impact by measuring anatomical pleiotropy, defined as the number of tissues or structures reported in the Zebrafish Information Network as affected by its genetic or experimental perturbation (Methods; Fig SCA in S1 Text). U genes display significantly higher anatomical pleiotropy than hS genes, but show no significant difference from S genes (Fig 4A). The proportion of genes with phenotypic annotations is similar for U and S genes (U: 2225/8579, ~ 26%; S: 1635/7551, ~ 22%), whereas hS genes have the lowest proportion (790/6446, ~ 12%). No systematic differences were observed among tissue-based subclasses within each developmental class (U-Ut, etc., Fig SCB in S1 Text).

To assess whether the high pleiotropy observed in these groups could be influenced by research bias, we examined the relationship between anatomical pleiotropy and publication frequency using ZFIN records (Fig SCC and SCD in S1 Text). Although the two are positively correlated (r = 0.69, p-value < 0.001), publication frequency is expected to reflect both annotation effort and biological factors, as genes with broad or essential functions often receive greater research attention [25]. An additional analysis using a uniformly annotated large-scale mutagenesis dataset indicates that, while annotation bias contributes to the observed relationship, a biological signal of anatomical pleiotropy remains detectable (Fig SCE in S1 Text). This result therefore supports interpreting the pleiotropy analysis as an exploratory measure that captures meaningful biological variation, while acknowledging that it is not completely independent of research intensity.

Because the stage of peak expression is a defining feature of gene classes during development (Fig 1D), we assessed its relationship to gene pleiotropy using a sliding-window approach (Methods). Both U and S genes follow a similar trajectory: pleiotropy increases from the zygotic stage, peaks at 6–8 hours post-fertilization (shield/epiboly during gastrulation), and then declines, suggesting that the timing of peak expression is a major determinant of pleiotropy (Fig 4B).

More specifically, U genes are enriched in a broader set of general anatomical terms (S4 Table; Fisher exact test: e.g., eye, head, liver) and are critical from gastrulation to early organ formation; their rapid decline in pleiotropy after the peak reflects simultaneous effects on multiple general structures. In contrast, S genes are enriched in fewer, more specific terms (e.g., portion of tissue, vein…) and sustain elevated pleiotropy over a longer developmental window, reflecting sequential effects on tissues and vascular structures. Finally, hS genes show relatively stable pleiotropy from 24 hpf to 3 dpf, followed by a decrease, and are consistently the least pleiotropic across all stages.

We also examined lethality, defined here as any genetic mutation that results in embryonic, larval, or adult death (an extreme manifestation of pleiotropy) and analyzed the distribution of 187 genes annotated as lethal. These genes are significantly enriched among U genes (97 of 8,579; hypergeometric test, p-value = 9.0e-06), but show no significant enrichment in either S genes (69 of 7,551) or hS genes (21 of 6,446,) (Fig 4C). In addition, lethal U genes predominantly peak at 2.25 hpf, with additional peaks at 0 hpf, 3 hpf (zygotic genome activation), and 6 hpf (gastrulation onset) (Fig SCF in S1 Text). Lethal S genes (>10%) peaks at 8–10 hpf, stages associated with the highest pleiotropy in S genes and not prominent in the overall S gene expression profile. In contrast, lethal hS genes peak later, mainly at 4 dpf, with secondary peaks at 3 hpf (as observed for some hS genes in Fig 1D) and 30 hpf during organogenesis (Fig S1F in S1 Text).

#### Phyletic age differs across developmental expression breadth classes.

**Fig 5:**
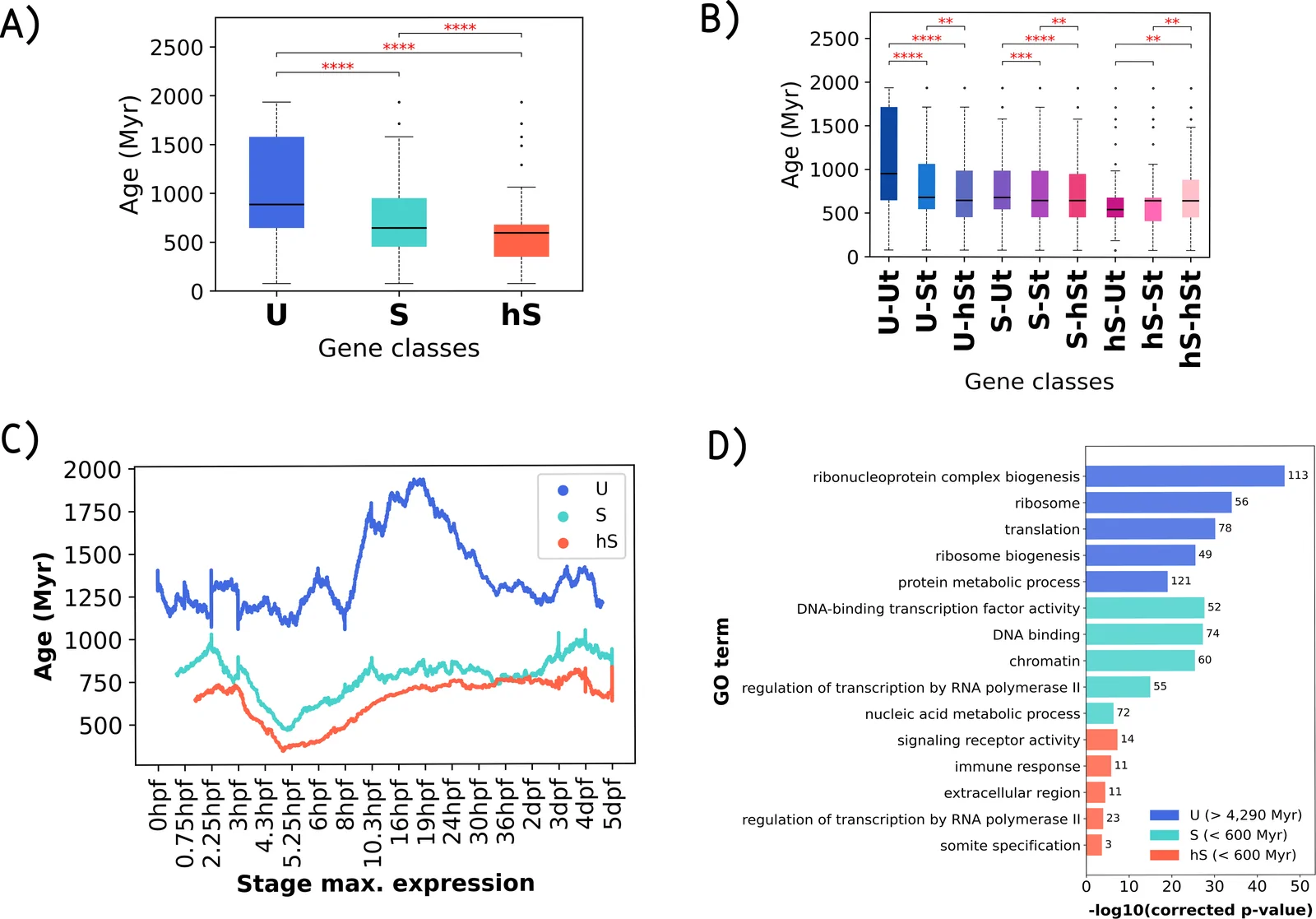
Analysis of phyletic age across development. **A)** Distribution of gene age per developmental class. All the distributions are significantly different; **** indicates a KS test p-value < 0.0001. We adjusted the y-axis limit and excluded outliers (genes with age > 4290 Myr, labeled as 4290) to improve visualization of the distributions. **B)** Distribution of gene age per developmental and tissue-based subclass. Y-axis limits and outliers were handled as in A). ** indicates a KS test p-value < 0.01; *** p-value < 0.001; **** p-value < 0.0001. Lack of asterisks denotes a non-significant p value. **C)** Gene age vs. the maximal stage of expression in bulk data. Sliding window = 300. **D)** Functional enrichment of the evolutionarily oldest U genes (> 4,290 Myr), which peak during the conserved zebrafish phylotypic stage (10.3–24 hpf), and of the youngest S and hS genes (< 600 Myr) that peak early in development (5.25–6 hpf). For each subset, enrichment was calculated using its corresponding main gene group as the statistical background. The y-axis shows selected GO terms with their associated gene counts, and the x-axis displays the -log10 of the corrected p-value.

Next, we examined phyletic age using the evolutionary age annotations available in GenOrigin (Methods). Given the general concordance between our developmental classification and *standard* HK genes, we expected that U genes would also be evolutionarily older, as reported for HK genes [26], and indeed, this was the case (Fig 5A; Methods), consistent with the idea that U genes encode core cellular functions under strong evolutionary constraint. Significant age differences were likewise observed among subclasses defined by adult tissue expression profiles (Fig 5B, note the qualitative difference in the U-Ut class).

To further examine how evolutionary age relates to gene expression dynamics across development, we next performed a sliding window analysis of age and peak expression per gene (Fig 5C, Methods). Note that in contrast to previous phylotranscriptomic approaches, our approach focuses on differences between gene classes across development rather than on global comparisons among stages, as in studies employing the transcriptome age index (TAI) [27,28]. A supplementary TAI-based analysis was performed to independently verify our approach (Fig SD in S1 Text; Methods).

Examining genes according to their stage of maximum expression revealed that, among S and hS genes, those peaking at approximately 5.25 hpf, immediately preceding gastrulation, have the youngest average phyletic ages. Conversely, among U genes, those peaking between 10.3 and 24 hpf—encompassing gastrulation, somitogenesis, neural tube formation, and the onset of organogenesis—have the oldest average phyletic ages. This pattern is consistent with the hourglass model of development, which posits that phylogenetically ancient genes tend to be expressed during the conserved mid-embryonic phase (the “phylotypic” stage), whereas younger, lineage-specific genes are more often expressed at earlier or later stages [17].

Given these expression-age patterns, we next examined the functions of the genes contributing to the most extreme stage-specific age profiles. Specifically, we analyzed ancient U genes (phyletic age > 4,290 Myr) reaching maximal expression between 10.3 and 24 hpf, corresponding to the conserved mid-developmental period (the zebrafish phylotypic stage) ([29]; Fig 5D and S5 Table). Using the remaining U genes as the background, these genes showed significant enrichment for core cellular processes related to ribosome biogenesis and protein synthesis, including ribonucleoprotein complex assembly and translation.

Conversely, we analyzed evolutionarily young S and hS genes (phyletic age < 600 Myr) reaching maximal expression between 5.25 and 6 hpf, the developmental interval associated with the youngest average phyletic ages within these classes (Fig 5D and S5 Table). Relative to the remaining genes in each class, S genes were enriched for nuclear processes, including transcriptional regulation, DNA binding, chromatin organization, and nucleic acid metabolism. hS genes shared many of these functions but additionally showed enrichment for immune response, receptor signaling, extracellular localization, and somite formation. These enrichments indicate that this subset of genes participates in regulatory processes associated with early developmental patterning.

Finer-grained tissue subclasses reveal additional distinctions. Within the U class, U-Ut genes exhibited the oldest phyletic ages (Fig 5B), with expression peaking between 10 and 19 hpf (Fig SEA in S1 Text). In contrast, U-St and U-hSt genes are significantly younger, with U-hSt genes—the youngest within the U class—peaking during blastulation and enriched for cell cycle–related processes, particularly cytoskeleton and microtubule dynamics. These findings are consistent with functions associated with proliferative processes that are broadly required during early development but become restricted to specific adult tissues.

Interestingly, subclass trends differ across gene classes. While the Ut subclass contains the oldest genes on average in both U and S categories (Fig SEA and SEB in S1 Text), this trend is reversed in the hS class (Fig SEC in S1 Text). hS-Ut genes, despite being classified as adult-tissue ubiquitous, are among the youngest in the dataset and are enriched for immune-related functions and responses to external stimuli (Fig 3F and S3 Table), likely reflecting the widespread distribution of immune cells across tissues. Together, these findings indicate that developmental expression breadth captures biologically meaningful differences that are reflected in evolutionary age and functional specialization.

#### Gene family histories reveal the split between housekeeping and stage-specific roles.

Finally, as the evolutionary age of a gene is closely associated with its family history, e.g., highly conserved genes typically possess numerous orthologs across species [30]. We now investigate gene homology across species, focusing on orthology between zebrafish and humans, and within species, examining paralogous relationships that emerge from gene duplication events in zebrafish (Methods).

**Fig 6:**
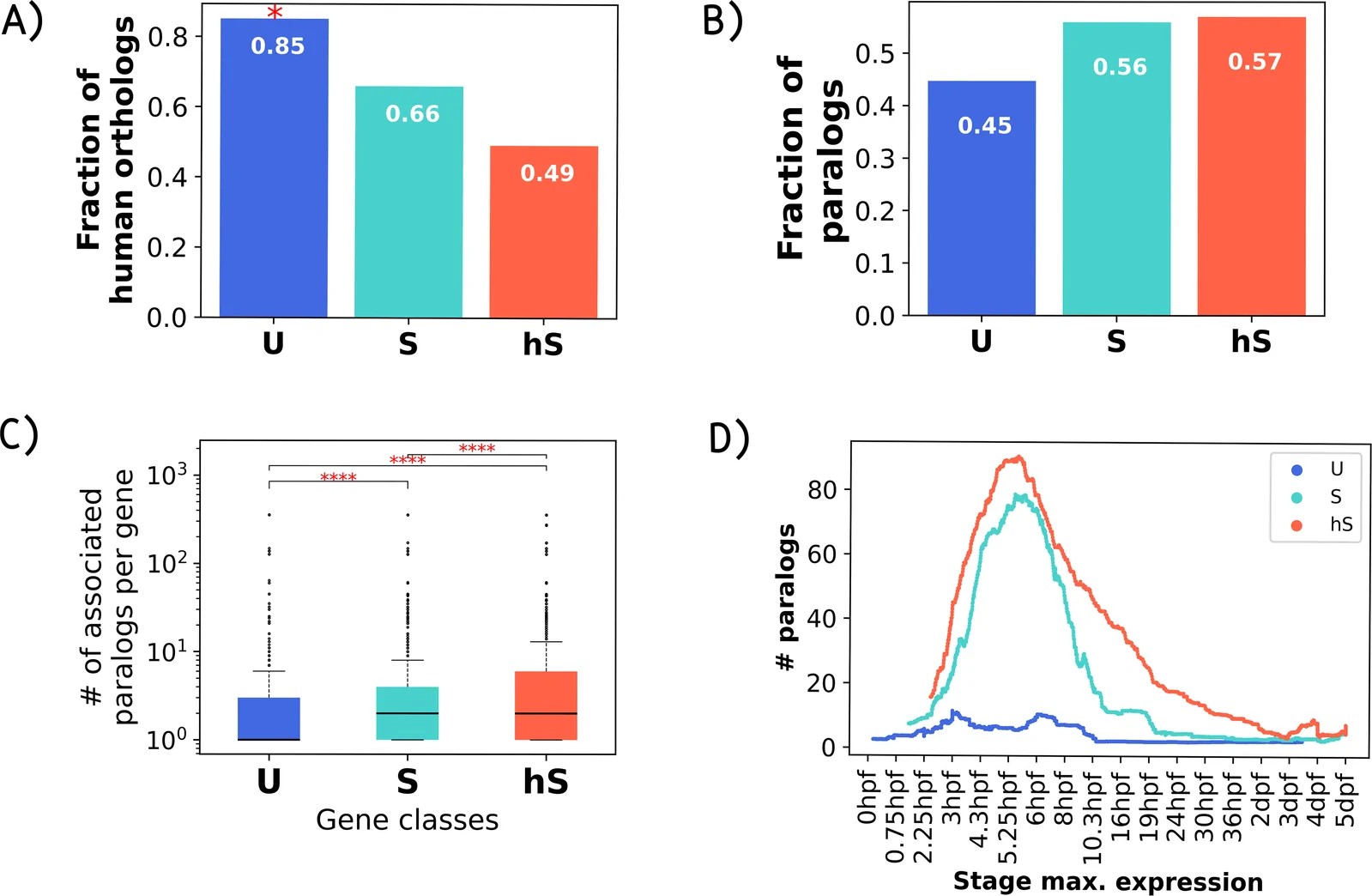
Homology analysis. **A)** Fraction of U, S, and hS genes with at least one human ortholog, relative to the total genes in each class. Asterisk indicates U class is significantly enriched based on a hypergeometric test (Methods, p-value < 0.0001). **B)** Fraction of U, S, and hS genes with at least one in-species paralog, relative to the total genes in each class. No class is significantly associated. **C)** Distribution of the number of paralogs per gene in each gene class. ** indicates a KS test p-value < 0.01; *** p-value < 0.001; **** p-value < 0.0001. **D)** Number of associated paralogs per gene vs. the maximal stage of expression in bulk data. Sliding window = 400.

Consistent with their ancient origin, the majority of U genes have human orthologs (85% – n = 7303; hypergeometric test, p-value < 2.225e^-308^), compared to 66% (n = 4980) of S genes and 49% (n = 3168) of hS genes (Fig 6A; see also Fig SFA in S1 Text for the distribution of orthologs across gene subclasses), aligning with our earlier findings on gene evolutionary age.

Moreover, paralogous genes, i.e., homologs within the same genome, drive functional innovation in multicellular organisms. Duplication followed by divergence allows them to adopt specialized roles, and prior studies show they are enriched in multicellular lineages and critical for expanding cell-type diversity [31]. Consistent with this, S and hS genes are more frequently associated with paralogs (S: 56% – n = 4223; and hS: 57% – n = 3672) than U genes (45% – n = 3835); however, no class shows significant enrichment (Fig 6B; see also Fig SFB in S1 Text for the distribution of paralogs across gene subclasses). Of note, the distribution of the number of paralogs per gene differs significantly across classes (Fig 6C).

To further characterise these patterns, we evaluated again how the number of paralogs varies across stages of peak expression using a sliding window analysis (Fig 6D). S and hS genes include subsets with a high number of paralogs that reach maximum expression between 5.25 and 8 hpf, during gastrulation. After this point, paralog numbers drop earlier in S genes (reaching low levels by ~10.3 hpf), whereas in hS genes the decline is more gradual, reaching similar values only by ~3 dpf. In contrast, U genes show a consistently low number of paralogs throughout development, with only a modest increase during gastrulation. These patterns further support the hourglass model, highlighting gastrulation as a stage where genetic redundancy may be particularly relevant and where innovation may transiently outweigh conservation.

Are there shifts between classes among paralogs? This may indicate regulatory divergence and the emergence of alternative developmental programs. Some pairs retain both genes within the same class (U–U: 32%, S–S: 51%, hS–hS: 41%), but the majority have at least one paralog assigned to a different class: 68% for U genes, 49% for S genes, and 59% for hS genes. These shifts are not random. Rather, paralogs often transition to the most adjacent class in terms of developmental expression breadth: U genes tend to have S-class paralogs, S genes most often pair with hS paralogs, and hS genes frequently have both S-class paralogs and paralogs that are not expressed during embryogenesis (S6 Table).

## Discussion

The concept of HK genes is typically framed as genes that are broadly expressed and support essential cellular functions. However, their identification in genome-wide studies has largely relied on measures of expression breadth across adult tissues. While these approaches have been instrumental in cataloging genes involved in core cellular processes, they effectively treat expression ubiquity as primarily a spatial property. A limited number of studies have additionally incorporated developmental transcriptomic data alongside adult datasets [12–14], but the relationship between *temporal* expression breadth during development and *spatial* expression breadth across adult tissues has not been systematically quantified. In this study, we directly compare these two dimensions, providing a framework to examine how developmental and tissue expression patterns jointly shape expression breadth.

Our results show that these two aspects are only partially concordant, revealing both shared and distinct gene classes. The subset of genes broadly expressed across both developmental stages and adult tissues represents a conserved core associated with fundamental cellular processes such as translation, RNA processing, and macromolecular biosynthesis. However, many genes classified as broadly expressed across adult tissues display restricted expression during embryogenesis, while some genes expressed consistently across development later become spatially specialized. Together, these findings demonstrate that expression breadth defined from adult tissues does not fully capture gene expression dynamics across the life cycle and underscore the importance of explicitly considering developmental time as an orthogonal dimension of expression diversity.

Importantly, our analysis treats broad expression as a distinct property from other commonly invoked characteristics of HK genes, such as high expression levels or low variability across conditions. This distinction may explain why different studies often identify only partially overlapping sets of these genes [7,11], as the resulting collections depend on both the transcriptomic datasets analyzed and the criteria used to define HK expression. Focusing on expression across diverse contexts is especially appropriate because it captures a defining feature while avoiding strong assumptions about quantitative expression levels, which can be influenced by technical and experimental factors such as sequencing depth, normalization strategy, and total RNA content. Although this definition still requires a detection threshold, it relies on relative presence or absence rather than precise quantification, making it less sensitive to these sources of variation and more closely aligned with the conceptual basis of HK genes. By prioritizing ubiquity over expression magnitude or stability, we clarify how this component of housekeeping-like behavior changes across the life cycle.

This focus on expression breadth not only clarifies the ubiquity component of HK-like behavior across the life cycle, but also provides a basis for testing whether developmental expression classes differ systematically in their functional, phenotypic, and evolutionary properties. Genes expressed across all developmental stages show strong enrichment for essential cellular processes and for phenotypes affecting multiple anatomical structures, consistent with high developmental pleiotropy. In contrast, genes with more restricted developmental expression are enriched for regulatory and signaling functions associated with developmental patterning and cell-type specialization.

These functional differences are also reflected in evolutionary patterns. Genes broadly expressed throughout development tend to be evolutionarily older than genes with more restricted expression, consistent with previous observations for HK genes. Moreover, within the developmentally ubiquitous class, genes with maximal expression during the phylotypic period exhibit the highest mean phyletic ages. This pattern is consistent with the developmental hourglass model, which proposes that mid-embryogenesis represents a period of maximal evolutionary constraint [17]. Together, these findings provide independent evolutionary support for the biological relevance of our developmental classification.

Consistent with this view, we also observe that genes expressed ubiquitously during development tend to have fewer paralogs than genes with more restricted expression. This pattern likely reflects stronger purifying selection and dosage sensitivity among genes involved in core cellular functions. In contrast, genes involved in regulatory and signaling pathways—more common in developmentally restricted classes—appear more permissive to duplication, consistent with the role of gene duplication in expanding regulatory complexity.

Because these biological differences are inferred from discrete expression classes, an important question is whether they depend on the thresholds used to define those classes. We adopted this strategy primarily for interpretability and to remain consistent with the conceptual distinction widely used in the literature between broadly expressed (“housekeeping”) and context-specific genes. Rather than forcing a binary classification, we defined three categories—ubiquitous (U), stage-specific (S), and highly stage-specific (hS)—to capture intermediate levels of developmental expression breadth. Importantly, the biological differences we report do not arise from a particular threshold choice. The three groups correspond to distinct regions of the underlying distribution of expression breadth, and show consistent, statistically significant differences in functional enrichment, phenotypic pleiotropy, and evolutionary properties. Moreover, similar trends are observed when expression breadth is treated as a continuous variable (Fig SG in S1 Text), indicating that our conclusions do not depend on discrete class boundaries. Likewise, varying reasonable detection thresholds yields the same qualitative patterns, suggesting that discretization serves as a convenient summary of continuous variation rather than a driver of the observed results (Fig SH in S1 Text).

At the same time, several limitations should be considered when interpreting these results. The developmental and adult tissue transcriptomes analyzed here are derived from bulk RNA sequencing of whole embryos, which cannot resolve cell-type–specific expression dynamics. Consequently, some genes classified as temporally ubiquitous may reflect shifting expression across distinct cell populations rather than continuous expression within individual cell types (Fig SI). Additionally, the developmental time series and the adult tissue atlas were generated independently using different experimental designs and quantification pipelines, and each captures only part of the full biological landscape with unequal sampling of the corresponding space. Consequently, the developmental and adult expression breadth categories should be interpreted as data-set derived representations of gene expression ubiquity in temporal and spatial contexts, rather than as definitive or universal classifications. Future studies integrating single-cell transcriptomic data and broader tissue sampling will enable a more refined characterization of multidimensional expression breadth.

Despite these limitations, our analysis highlights the value of considering both developmental and adult transcriptomic data when examining gene expression breadth. More broadly, our findings support the view that developmental and adult expression-based definitions provide complementary perspectives on gene expression ubiquity across the life cycle. Examining developmental expression breadth and its temporal dynamics reveals biologically meaningful relationships with functional roles and evolutionary constraints.

## Methods

### Bulk RNA-seq data along development

The mRNA expression time-course data for zebrafish (*Danio rerio*) development were obtained by [15]. This data includes transcriptomic profiles across 18 developmental stages, from the one-cell stage to five days post-fertilization. At each stage, transcriptomes were measured from five independent biological replicates, with each replicate comprising a pool of 12 embryos.

We downloaded the transcript abundance matrix provided in the Supplementary Materials of the original publication, expressed as Transcripts Per Million (TPM). For each developmental stage, we calculated the mean expression across the five biological replicates to obtain a representative expression profile (<TPM>). Because averaging compositional data does not preserve the constant-sum constraint of the original samples, the averaged profiles were subsequently renormalized to the standard TPM scale before applying the expression threshold used in downstream analyses.

### Bulk RNA-seq data per tissue (adult zebrafish)

Tissue mRNA expression data for *Danio rerio* were obtained by [16]. The dataset is available in the Gene Expression Omnibus (GEO) repository (www.ncbi.nlm.nih.gov/geo) under accession code GSE62221. Transcriptomic profiles were measured across eight tissues (brain, gill, heart, intestine, kidney, liver, muscle, and spleen) under three temperature conditions: 28°C, 18°C, and 10°C. The original expression matrix was reported in Reads Per Kilobase of transcript per Million mapped reads (RPKM). For our analysis, we transformed the data into Transcripts Per Million (TPM). Only data at 28ºC were analyzed in this work.

### Single-cell RNA-seq data along development

Developmental single-cell RNA-seq (scRNA-seq) data for *Danio rerio* were obtained from the Zebrahub dataset [32], available at https://zebrahub.sf.czbiohub.org/data. This comprehensive dataset encompasses 10 developmental stages, ranging from end-of-gastrulation embryos to 10-day-old larvae. For each time point, four embryos were sequenced, yielding a total of approximately 120,440 analyzed cells.

### Single-cell RNA-seq data for adult zebrafish

Adult single-cell transcriptomic data were obtained from the Zebrafish Cell Landscape (ZCL) version 2.0 [33], accessible at http://bis.zju.edu.cn/ZCL/. While the complete ZCL 2.0 dataset comprises over 1,360,000 single cells analyzed across five zebrafish life stages, our analysis exclusively utilized data from the 22-month-old adult stage. Because the original data were provided as a Seurat object, raw count matrices and cell metadata were extracted using R to ensure compatibility with our analytical pipeline. These data were subsequently assembled into an AnnData format for downstream analysis in Python.

### Gene classification by tissue specificity and comparison with developmental classes

Using bulk RNA-seq tissue data (see Tissue Data), we classified genes based on the number of tissues in which they were expressed (TPM > 1). We defined three categories: ubiquitous tissue (Ut) genes, expressed in all analyzed tissues; tissue-specific (St) genes, expressed in >50% of tissues; and highly tissue-specific (hSt) genes, expressed in <=50% of tissues. We then intersected this tissue-based classification with the developmental gene classes (U, S, hS) and quantified the distribution of each developmental class across the Ut, St, and hSt categories.

Although developmental and adult tissue datasets were converted to TPM to facilitate comparison and a common expression threshold (TPM > 1) was applied consistently, differences in sample preparation, sequencing, transcript quantification, and preprocessing may influence the classification of genes across datasets. Consequently, the developmental and adult expression breadth categories should be interpreted as an approximate framework for comparing temporal and spatial dimensions of gene ubiquity, rather than as an absolute equivalence between the two.

### Data availability for gene–phenotype associations and lethality annotations

Gene–phenotype association data were downloaded from ZFIN (The Zebrafish Information Network, https://zfin.org/; Data Reports, 26 Jan 2025), the primary database for genetic and genomic information on zebrafish (*Danio rerio*). The ontology organizes phenotypes using anatomical terms, specifying which structures or tissues are affected by genetic or experimental manipulations (see Supplement).

To quantify anatomical pleiotropy, we counted the total number of distinct anatomical terms associated with each gene, regardless of the developmental stage in which they appeared, noting that the number of terms increases with developmental complexity (Fig SCA in S1 Text).

Lethality information was also extracted from ZFIN. Genes annotated with the phenotype ‘lethal (sensu genetics)’ were retrieved, resulting in 329 ZFIN gene entries. After mapping to Ensembl identifiers, 190 genes were retained, of which 187 were present in our bulk developmental RNA-seq dataset.

### Data availability for the phyletic age section

Phyletic age assignments were obtained from GenOrigin (http://genorigin.chenzxlab.cn/), a database that provides inferred evolutionary ages for genes based on comparative genomic analyses across multiple species [34]. The reported ages correspond to inferred ancestral phylogenetic nodes expressed as approximate divergence times (Myr), rather than continuously estimated gene ages. Consequently, they should be interpreted as broad relative evolutionary age assignments, with a resolution determined by the underlying phylogenetic sampling. Some genes were assigned the maximum age category (>4290 Myr). For numerical analyses, these genes were assigned a value of 4290 Myr, corresponding to the upper age limit represented in the database.

### Data availability for the gene homology section

Orthologous gene pairs between zebrafish and human were obtained from the ZFIN database (https://zfin.org/downloads). Specifically, the orthology data were retrieved from the “Human and Zebrafish Orthology” CSV file available under the “Orthology Data” section of the Downloads page. Zebrafish paralogs were retrieved from Ensembl BioMart (https://www.ensembl.org/biomart/martview) by selecting the *Danio rerio* dataset and the “Paralogues” option under Homology attributes.

### Data availability for the transcriptional mechanism section

Zebrafish transcription factors and cofactors were retrieved from Animal TFDB4 (https://guolab.wchscu.cn/AnimalTFDB4/#/). From 2,546 transcription factors, we identified 1,973 in our data. From 782 cofactors, we identified 688.

### Temporal and spatial expression specificity

To quantify temporal (developmental stages) and spatial (tissues) specificity, we use the Yanai tissue specificity index, τ [18], calculated as:


$$\begin{array}{c} \tau =\frac{\sum_{i=\text{1}}^{N}(\text{1}-\frac{x_{i}}{x_{max}})}{N-\text{1}}\;, \end{array}$$

where N is the total number of developmental stages or tissues evaluated, $x_{i}$ is the expression level of the gene in a specific stage or tissue i, and $x_{max}$ is the maximum expression level of that gene across all stages or tissues.

### Temporal and spatial expression variability

To measure the temporal and spatial expression variability, we calculated the coefficient of variation (CV) for each gene across all stages and tissues:


$$\begin{array}{c} CV=\frac{\sigma }{\mu }, \end{array}$$

where μ represents the mean expression of the gene across all evaluated stages or tissues, and σ represents the standard deviation of its expression.

### Adjusted coefficient of variation

The adjusted coefficient of variation (CV) was calculated following [35], to obtain a mean-independent CV. For each gene, we computed the CV at each stage using TPM values from the five biological replicates per stage and then transformed it as log_10_(CV²). Genes were ordered by overall mean expression, and for each gene, we subtracted the median log_10_(CV²) of 50 neighboring genes with similar expression. Positive values indicate that a gene is more variable than genes with comparable expression, while negative values indicate greater stability.

### Calculation of the stage of maximal expression

For each gene, we identified the developmental stage at which its expression reached the maximum value across all stages. Then, for each gene class, we calculated the percentage of genes showing maximal expression at each stage. This analysis yielded the distribution of maximal expression stages for each gene class (Fig 1D).

### Hypergeometric test

We used the hypergeometric test to assess whether specific gene classes were significantly enriched in functional categories, such as transcription factors, cofactors, or essential genes. The background set consisted of all genes in the bulk dataset, while the gene sets of interest included all genes associated with our data. For each class (U, S, hS) and their respective subpopulations, the test calculates the probability of observing the overlap with a functional category by chance, using the hypergeometric distribution. This approach determines whether the representation of genes in each category is greater than expected randomly.

### Sliding window

To identify patterns between two biological variables, we applied a sliding window approach using Python’s Pandas library, specifically the rolling().mean() method. Genes were first sorted by their stage of maximal expression, establishing the x-axis (developmental trajectory). The corresponding values of the y-axis (the gene-associated magnitude) were then reordered to match this sorted trajectory. A sliding window of size *sw* was applied to both axes to compute a rolling average. For example, the first computed average corresponds to values from x[0] to x[*sw*-1]. The next window includes values from x[1] to x[*sw*], then x[2] to x[*sw* + 1], and so on. This process continues until the end of the array. Positions with fewer than sw values return NaN, ensuring all averages are computed from complete windows. This technique reduces noise and highlights underlying trends that may not be apparent in the raw data.

### Gene Ontology enrichment analysis

We downloaded Gene Ontology (GO) annotations from Gene Ontology Consortium (format-version: 1.2, data-version: releases/2025-02-06). We built three gene-GO term association matrices one for each aspect of the ontology (molecular function, cell component and biological process), by expanding direct annotations to ancestor terms in the GO hierarchy. GO term enrichment was assessed for each gene subset (U, S, hS) and their derived subsets (e.g., U-Ut, U-St) using Fisher’s exact test (fisher_exact function from scipy.stats), comparing the respective gene set against the complementary set comprising all remaining genes in the dataset. We corrected p-values within each GO aspect for multiple testing using False Discovery Rate (FDR).

### Continuous GO enrichment analysis

To assess GO term enrichment with respect to the continuous temporal specificity metric (τ), we applied a distribution-based approach. For each GO term, genes were divided into two groups: those annotated to the term and those not annotated. We compared the distributions of τ values between these groups using a two-sample Kolmogorov–Smirnov (KS) test. For terms with significant differences, we calculated the median τ difference between annotated and non-annotated genes. This difference was used to determine enrichment direction, classifying GO terms as associated with either low τ (ubiquitous expression) or high τ (specific expression).

### Transcriptome Age Index (TAI)

To verify our phylotranscriptomic analyses, we calculated the TAI [27] across development by mapping gene origins to 22 discrete evolutionary nodes (spanning >4290–76 million years ago). We ranked these nodes chronologically, assigning the most ancient origin (>4290 mya) to Phylostratum 1 and the youngest (76 mya) to Phylostratum 22. The index for each developmental stage (s) was computed as:


$$\begin{array}{c} {TAI}_{s}=\frac{\sum_{i=\text{1}}^{n}\left(e_{i,s}\;\cdot {\;ps}_{i}\right)}{\sum_{i=\text{1}}^{n}e_{i,s}} \end{array}$$

where ${\;ps}_{i}$represents the phylostratum rank of gene i, $e_{i,s}$ denotes the expression level of gene i at developmental stage s, and n is the total number of genes analyzed.

## Supporting information

S1 Text Supplementary analyses, Figs SA–SI and Table SA. (PDF)

S1 Table List of genes classified across development using RNA-seq data obtained by White et al. [15] and across tissues using RNA-seq data obtained by Hu et al. [16]. (XLSX)

S2 Table Gene Ontology (GO) enrichment of the three gene classes across development: U, S, and hS. (XLSX)

S3 Table GO enrichment of gene subsets: U-Ut, U-St, U-hSt, S-Ut, S-St, S-hSt, hS-Ut, hS-St, and hS-hSt. (XLSX)

S4 Table Enrichment of anatomical terms from ZFIN for U, S, and hS genes. (XLSX)

S5 Table GO enrichment analyses of gene subsets defined by maximal expression stage and phyletic age. Subsets of ancient U genes (phyletic age > 4,290 million years) with peak expression at stages 10.3, 16, 19, and 24 hpf, and subsets of young S and hS genes (phyletic age < 600 million years) with peak expression at stages 5.25 and 6 hpf were included.
(XLSX)

S6 Table Classification analysis of paralog gene pairs, evaluating whether both genes in each pair are classified within the same developmental class or in different classes. (XLSX)

S7 TableGO enrichment analysis of the continuous temporal specificity metric (τ).(XLSX)
