# Supplementary material for: Developmental and tissue expression breadth define distinct but overlapping classes of housekeeping genes
Source: PLoS Comput Biol. 2026 Sep 21;22(9):e1014787. doi: 10.1371/journal.pcbi.1014787 (PMC13607803; doi:10.1371/journal.pcbi.1014787)
Supplement: Supplementary file 1 [file pcbi.1014787.s001.pdf]

Supplementary material

Developmental and tissue expression breadth define distinct but overlapping classes of housekeeping genes

Alicia Lou[1], Juan F Poyatos[2], Monica Chagoyen[3]

[1] Centro Nacional de Biotecnologia (CNB-CSIC)

[2] Museo Nacional de Ciencias Naturales (MNCN-CSIC)

[3] Centro de Neurociencias Cajal (CNC-CSIC)

Classification of genes by their developmental expression: justification of discrete gene classes

We introduced a classification of genes by their developmental expression (Fig SAA in S1 Text) and analyzed the continuous distribution of the fraction of stages detected across all expressed genes (Fig SAB in S1 Text). Partitioning this distribution into tertiles showed that the 33rd and 66th percentiles occur at 9 stages (fraction = 0.50) and 18 stages (fraction = 1.0), respectively. Our discrete cutoffs align exactly with these data-driven boundaries: the hS class (≤ 8 stages) captures the lower tertile, the U class (18 stages) isolates the upper tertile, and the S class (9–17 stages) represents the intermediate tertile. Thus, the classification reflects the intrinsic structure of the developmental continuum.

To validate discrete classification bins against continuous expression profiles across development, we calculated temporal specificity (τ) (Yanai et al., 2005)and expression variability (CV) using log(TPM+1) values (Fig SAC and SAD in S1 Text; Methods). Both metrics demonstrated a strong negative correlation with the fraction of detected developmental stages (Pearson's r=-0.88 for τ; r=-0.86 for CV; p-value<0.001). Consistent with biological expectations for temporal expression, highly specific (hS) genes exhibited the greatest variability and specificity, while ubiquitous (U) genes showed the lowest. Notably, these ubiquitous genes still maintained non-zero CV and τ values, indicating that their baseline expression fluctuates across different developmental stages despite being broadly detected.

To externally validate our developmental classification, we repeated the categorization using bulk RNA-seq data from Bo et al., (2025). The following analysis compares the assignment of

genes to the Ubiquitous (U), Specific (S), and Highly Specific (hS) categories between the White et al., (2017) and Bo et al., (2025) datasets.

The Bo et al., (2025) dataset, comprising bulk transcriptomic data across 21 developmental stages (in triplicate), was processed by averaging biological replicates and strictly re-normalizing each stage to exactly 1,000,000 TPM. Active gene expression was defined using a TPM > 1 threshold, so genes below this threshold are considered Non-Expressed (NE). To ensure a strict 1:1 temporal alignment with the 18 stages evaluated in the White et al. (2017) study, the Bo et al. timeline was filtered by excluding the *unfertilized* and *oblong* (~3.6 hpf) time points (both omitted in White et al.), and the *day 6* time point, since the timeline concludes at 5 dpf.

Importantly, to independently validate our classification scheme, we directly applied the exact stage-based cutoffs defined from the White et al. dataset (hS: ≤ 8, S: 9–17, U: 18 stages) rather than recalculating the tertile boundaries for the Bo et al. dataset. Because of dataset-specific differences, an independent tertile partition in the Bo et al. data would have shifted the stage divisions. By freezing these stage thresholds, we were able to strictly assess the reproducibility of the categorical definitions themselves.

Our comparison of the genes present in both datasets reveals a highly conserved classification of developmental expression dynamics (Fig SAE in S1 Text):

- **U genes** (total in Bo et al.: 8,228): Of the 7,947 U genes that overlap with the White et al. dataset, 96.09% maintained their Ubiquitous classification. The remaining genes were previously classified as S (3.69%), hS (0.18%), or NE (0.05%).
- **S genes** (total in Bo et al.: 7,991): Of the 6,298 S genes overlapping with the older dataset, 85.85% retained their Specific classification. The rest fluctuated slightly to hS (7.19%), U (6.27%), or NE (0.68%).
- **hS genes** (total in Bo et al.: 4,008): Of the 2,986 overlapping hS genes, 84.93% were also classified as hS in the White et al. dataset. The remainder shifted to S (11.55%), NE (3.28%), or U (0.23%).
- **NE genes** (TPM < 1) (total in Bo et al.: 570): Among the 182 overlapping genes classified as unexpressed in Bo et al., 54.95% were also NE in the White et al. dataset, while the rest were previously detected at low specificities (36.81% hS, 6.59% S, 1.65% U).

These results demonstrate the high reproducibility of our temporal categorization, as most genes strictly retain their developmental class across independent datasets. The minor classification shifts observed are expected variations arising from differences in experimental design, sequencing depth, and transcriptomic technologies.

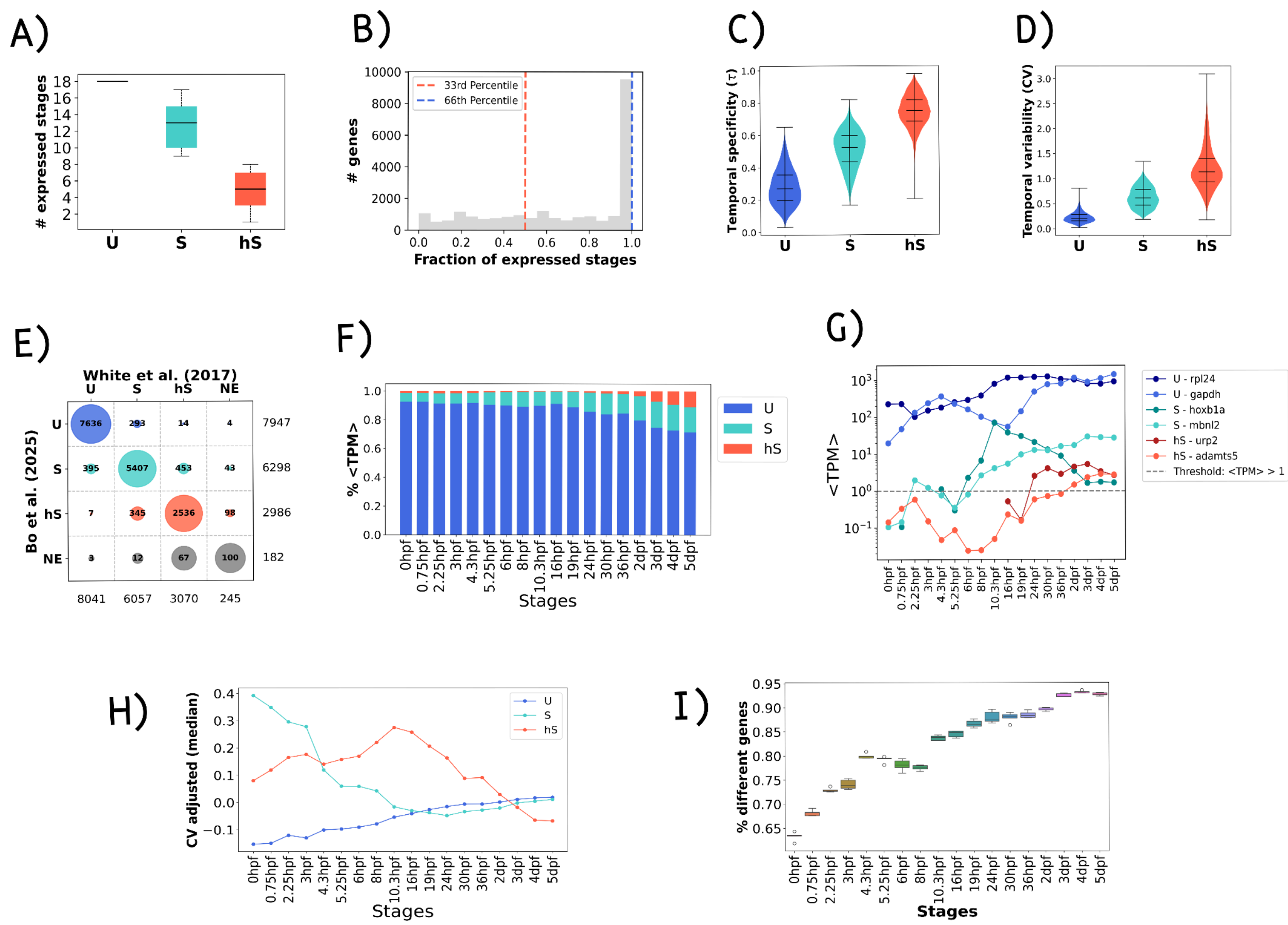

Fig SA. Classification of genes by their developmental expression. We grouped genes based on their developmental expression profiles (obtained by White et al., 2017) by quantifying the number of stages in which each gene is expressed. The dataset includes 18 stages (hpf = hours post-fertilization; dpf = days post-fertilization). Gene expression was established using a threshold of TPM > 1. Ubiquitous (U) genes are represented in dark blue, specific (S) genes in light blue, and highly specific (hS) genes in red. **A)** Distribution of the number of stages in which a gene is expressed, by gene class. **B)** Histogram showing the continuous distribution of expressed stages per gene. Dashed lines denote the 33rd (9 stages, fraction = 0.50) and 66th (18 stages, fraction = 1.0) percentiles. Our discrete cutoffs—hS (≤ 8), S (9–17), and U (= 18)—perfectly mirror these statistical tertiles. **C)** Violin plot distribution of temporal specificity (τ) per gene class (see Methods). **D)** Violin plot distribution of temporal variability (coefficient of variation, CV) per gene class (see Methods). **E)** Comparison of White et. al (2017) and Bo et al. (2015) developmental classifications. Points show the proportion of genes in each class of the Bo dataset assigned to each category in the White dataset, with point size indicating the row-wise percentage. Most genes conserve their class in both developmental datasets. Non-Expressed (NE) genes (those with TPM < 1) are represented in gray. **F)** Proportion of total transcript abundance (TPM > 0) accounted for by each gene class at each developmental stage. **G)** Average expression (<TPM>) of two representative genes for each gene class (U, S, and hS). The gray dashed line indicates the threshold used to consider a gene as actively expressed in each stage. Missing data points correspond to stages with no detectable

expression. **H)** Average stability of genes per class at each stage, measured as the adjusted CV per gene class (see Methods). Values are based on TPM from five biological replicates; negative values indicate lower variability than genes with similar mean expression. **I)** Fraction of expressed genes per developmental stage. A gene was considered expressed if TPM > 0. For each developmental stage, five biological replicates were processed independently, and the fraction of expressed genes was quantified separately for each replicate. Thus, each boxplot represents the distribution of these five replicate-level fractions. Note that each biological replicate is a pool of 12 embryos.

Classification of genes by their tissue expression: justification of discrete tissue gene classes

We analyzed the continuous distribution of the fraction of tissues detected across all expressed genes (Fig SBB in S1 Text). Partitioning this distribution into tertiles showed that the 33rd and 66th percentiles occur at 4 tissues (fraction = 0.50) and 8 tissues (fraction = 1.0), respectively. Our discrete cutoffs align exactly with these data-driven boundaries: the hS class (≤ 4 stages) captures the lower tertile, the U class (8 tissues) isolates the upper tertile, and the S class (5–7 tissues) represents the intermediate tertile. Thus, the classification reflects the intrinsic structure of the developmental continuum.

To validate discrete classification bins against continuous expression profiles across tissues, we calculated spatial specificity, τ and expression variability (CV) using log(TPM+1) values (Fig SBC and SBD in S1 Text; Methods). Both metrics showed strong negative correlations with the fraction of detected tissues (r=-0.86 for τ; r=-0.85 for CV; p-value<0.001).

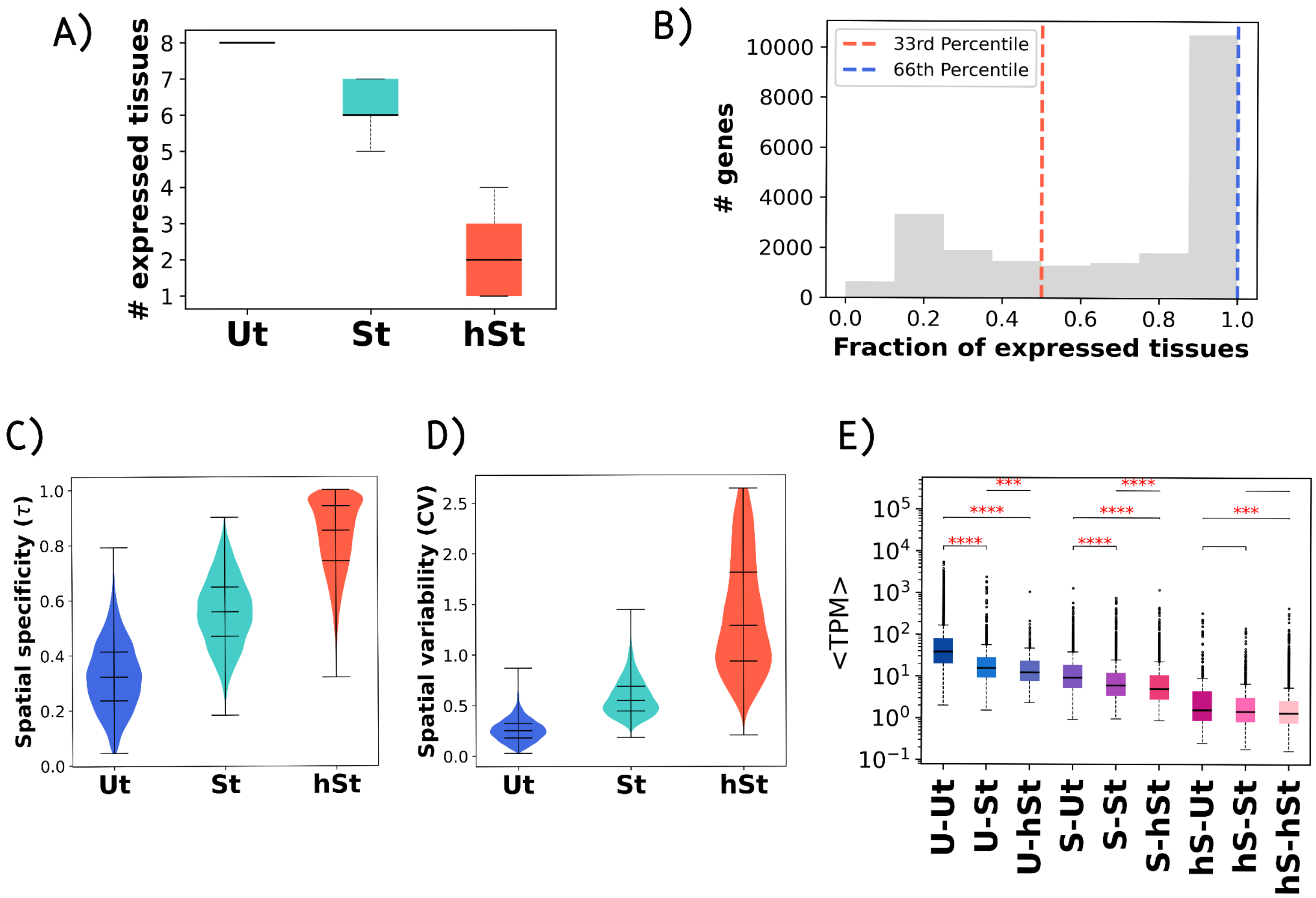

Fig SB. Gene classification based on tissue specificity in adult tissues. We grouped genes based on their tissue expression obtained by (Hu et al., 2015)**. A)** Distribution of the number of tissues in which a gene is expressed, by gene class. **B)** Histogram showing the continuous distribution of expressed tissues per gene. Dashed lines denote the 33rd (4 tissues, fraction = 0.50) and 66th (8 tissues, fraction = 1.0) percentiles. Our discrete cutoffs—hSt (≤ 4), St (5–7), and Ut (= 8)—perfectly mirror these statistical tertiles. **C)** Violin plot distribution of spatial specificity (τ) per gene class (see Methods). **D)** Violin plot distribution of spatial variability (coefficient of variation, CV) per gene class (see Methods). **E)** Distributions of mean expression levels along development, calculated by averaging developmental stages where genes showed detectable expression (TPM > 0). Subcategories within developmental classes were distinguished based on tissue specificity. Kolmogorov–Smirnov (KS) tests were performed between distributions belonging to the same developmental class. *** indicates a KS test p-value < 0.001; **** p-value < 0.0001. Lack of asterisks denotes a non-significant p-value.

Genotype–Phenotype associations, heterogeneity of perturbations, and study bias

Phenotype annotations from ZFIN (The Zebrafish Information Network, https://zfin.org/ ; Data Reports, 26 Jan 2025, 'Phenotype of Zebrafish Genes') derive from several types of genetic manipulations. To address potential heterogeneity in perturbation types, we systematically

classified the underlying manipulations for the 158,687 gene-phenotype associations in our dataset based on standard ZFIN genotype nomenclature (e.g., identifying stable alleles via superscript notation, morpholinos via 'MO', and transgenes via 'Tg').

Programmatically, this classification was performed by parsing the 'genotype' column: morpholinos were prioritized if the string contained 'MO'; pure stable mutations if it contained solely the superscript notation ('<sup>'); stable mutants with transgenes if it contained both '<sup>' and 'Tg'; pure transgenics if it contained only 'Tg'; and the rest as unclassified or wild-type backgrounds. We found that the majority of our data stems from stable, heritable mutations where observed phenotypes directly reflect long-term gene loss-of-function. Specifically, 45.95% of associations correspond to pure stable mutations (homozygous/heterozygous) and 10.65% to stable mutants combined with transgenes. Morpholino-mediated knockdowns account for 40.36%, while pure transgenics (1.15%) and transient/unclassified edits (1.89%) constitute a minority. We recognize that combining these techniques introduces noise: morpholinos frequently overestimate pleiotropy due to off-target toxicity, whereas stable mutants can underestimate it due to transcriptional adaptation (genetic compensation). Nevertheless, we retained all categories because excluding non-stable perturbations would severely reduce the biological depth of the dataset and discard thousands of valid annotations.

To address the effect of research bias, we examined the relationship between anatomical pleiotropy and publication frequency (obtained from ZFIN; each gene-phenotype association is linked to a publication; Fig SCC and SCD in S1 Text). Although these variables are correlated ($r = 0.69$, p-value < 0.001), this also reflects a biological pattern whereby genes with broad, essential functions attract greater research attention (Lou et al., 2026; Stoeger et al., 2018). Using regression analysis, we identified genes whose research intensity deviates from phenotypic annotations. We found that the top outliers (highest residuals) consist of genes with a vast publication record but relatively low anatomical pleiotropy. Despite being 'over-studied' relative to the average, these genes do not show the broad anatomical associations expected by the regression model.

To definitively disentangle biological pleiotropy from study intensity, we recalculated pleiotropy using a dataset with uniform annotation effort. We identified those publications reporting phenotypes for >100 distinct genes, assuming these represent unbiased, large-scale screens. This criterion yielded only two records: a bulk automated curation file (ZFIN Historical Data, 2006), which we excluded, and a single large-scale insertional mutagenesis screen encompassing 315 embryonic-essential genes and 114 phenotypes (Amsterdam et al., 2004; ZDB-PUB-040708-1).

Using this isolated, uniformly annotated dataset, we calculated unbiased pleiotropy scores and compared them to the overall scores from our full, heterogeneous database (Fig S3E in S1 Text). The two metrics showed a low but significant positive correlation (Pearson's $r = 0.145$, p-value < 0.01). This demonstrates that while cumulative annotation bias inflates global pleiotropy estimates, a genuine biological signal of pleiotropy persists. However, because this stringent filtering eliminates the vast majority of genes and complex late-stage phenotypes,

completely removing research bias is impossible without severely sacrificing the biological depth of the dataset.

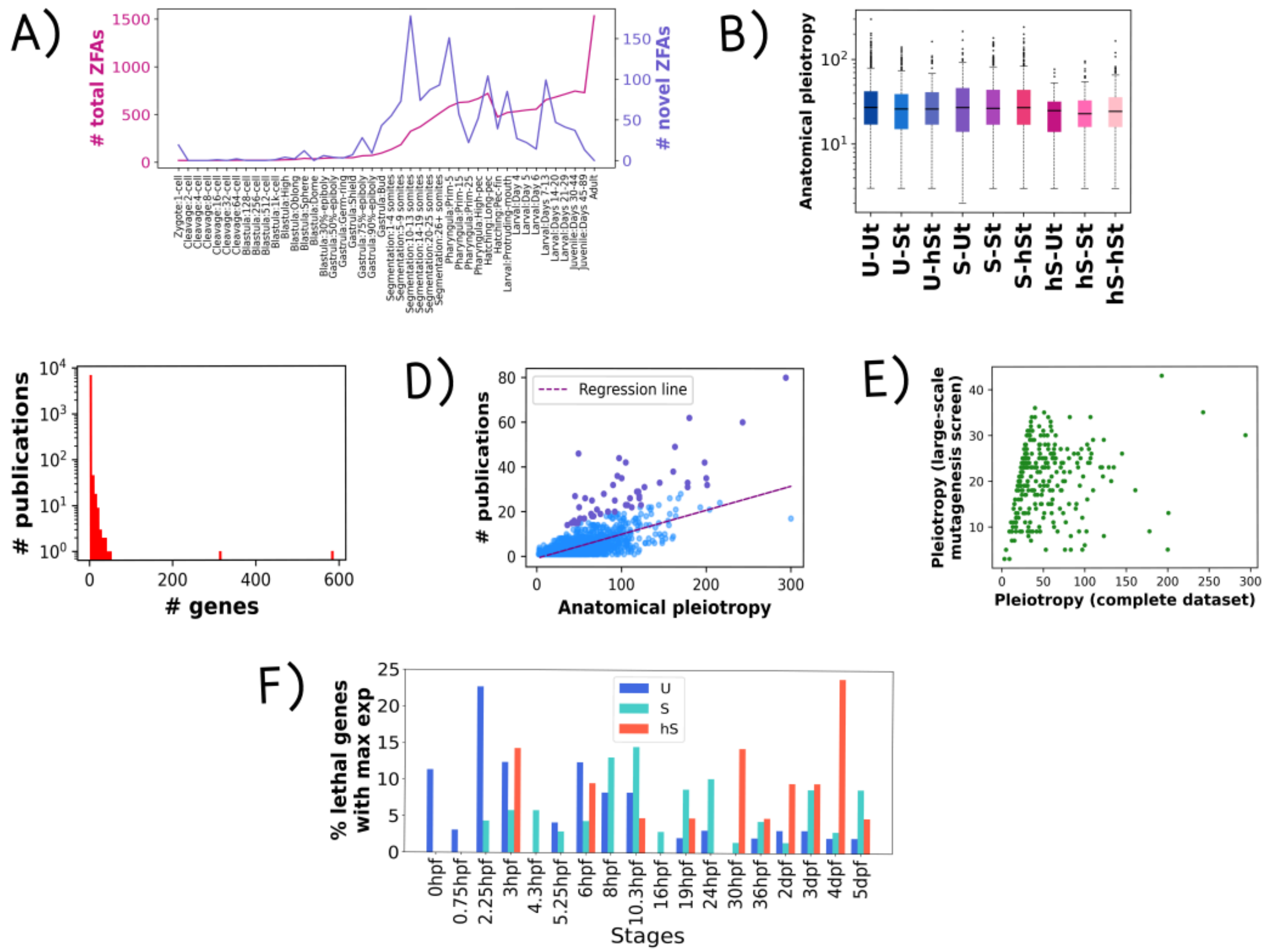

Fig SC. Anatomical pleiotropy analysis. A) Complexity of zebrafish and gene expression along development. Novel (purple) and total (magenta) anatomical phenotypes (ZFA: Zebrafish Anatomy Ontology) per developmental stage. Zebrafish anatomical complexity generally increases during development. However, temporary structures can cause fluctuations, such as a slight decrease after hatching. Data obtained from ZFIN. **B)** Anatomical pleiotropy distribution among developmental - tissue specificity classes. No significant differences were found within the same developmental classes. **C)** Distribution of the number of genes associated per publication. Data is derived from ZFIN gene-phenotype associations, representing the count of unique genes linked to each study. Notably, only two publications are associated with more than 100 distinct genes. **D)** Number of publications versus anatomical pleiotropy. The purple line represents the linear regression, indicating the expected number of publications for a given level of pleiotropy. The majority of outliers (purple points, representing the highest residuals) fall above the regression line, indicating genes that have a higher-than-expected number of publications based on their anatomical pleiotropy. **E)** Comparison of anatomical pleiotropy per gene. This compares the pleiotropy of genes from the remaining publication with >100 associated genes against the complete dataset. The single publication with the highest overall number of genes was excluded from this specific analysis, as it represents the core ZFIN database itself. **F)** Stages of maximal expression for lethal genes in each gene class. Shown is

the percentage of lethal genes within each class (relative to the total number of lethal genes in U, S, and hS classes) whose maximal expression occurs at each developmental stage.

Transcriptome Age Index analysis

To validate our phylotranscriptomic analyses, we calculated the Transcriptome Age Index (TAI; Domazet-Lošo & Tautz, 2010; see Methods) across developmental stages by mapping gene origins to 22 evolutionary nodes (>4,290 to 76 Myr). The global TAI, incorporating all gene classes (U, S, and hS), successfully recapitulated the transcriptomic hourglass model (Fig SDA in S1 Text). The most evolutionarily conserved profile (lowest TAI) occurred during mid-embryogenesis (10.3–24 hours post-fertilization [hpf]), with maximum conservation at 16 hpf. To determine the contribution of each gene class to this global pattern, we performed a cumulative TAI analysis (Fig SDB in S1 Text). U genes primarily drive the hourglass dynamic. The addition of S genes subtly refines this profile, whereas hS genes have negligible impact on the global TAI due to their low expression levels. Finally, computing class-specific TAIs revealed the internal evolutionary dynamics of each group (Fig SDC in S1 Text). U genes consistently exhibited the lowest TAI, indicating a strong reliance on ancient genes. Conversely, hS genes displayed the highest TAI, reflecting the recruitment of younger genes. Notably, the hS age index peaked between 4.3 and 8 hpf, highlighting the prominent transcriptional role of evolutionarily young genes during these early stages, consistent with our prior age analyses in the main text.

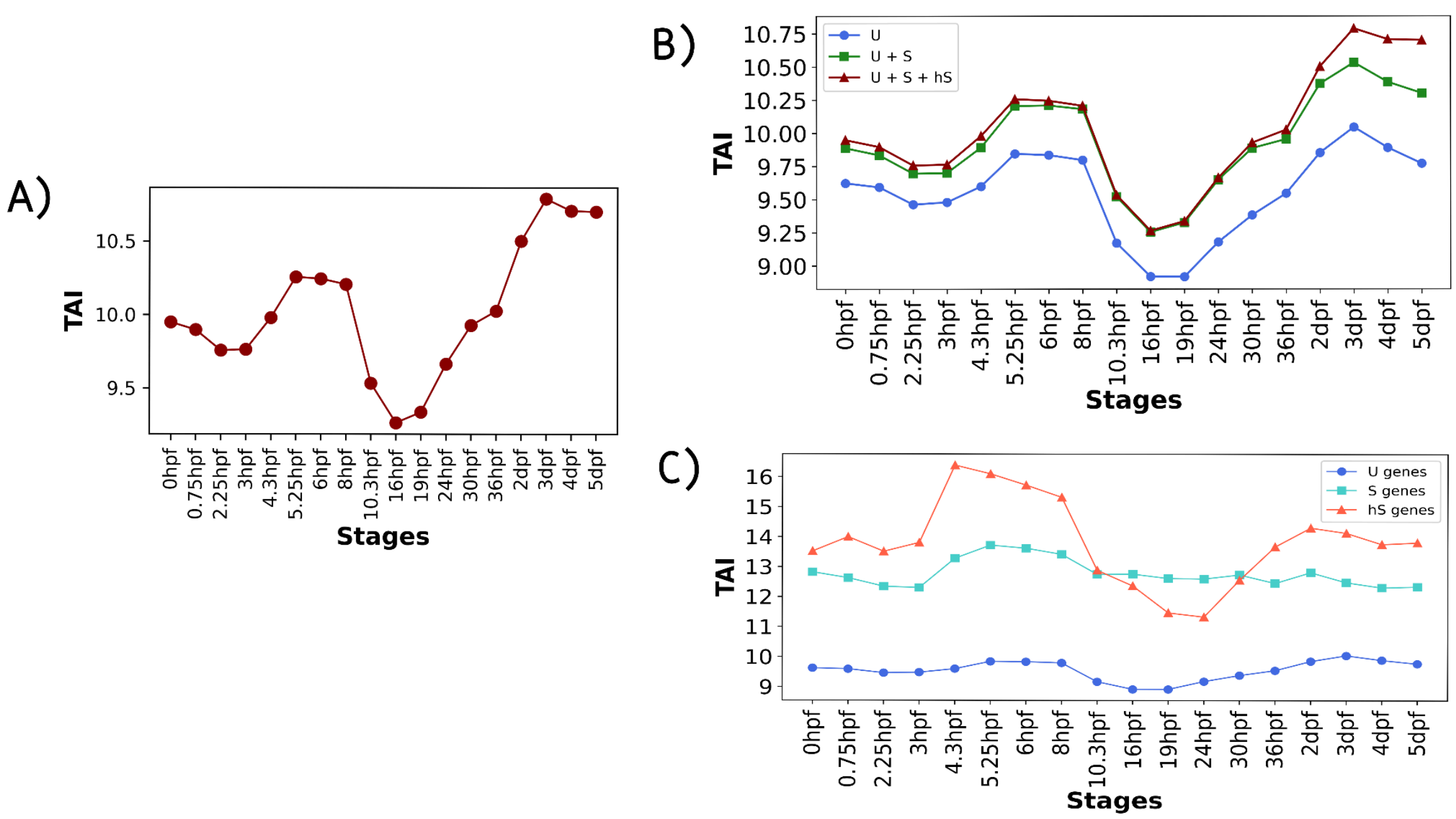

Fig SD. Transcriptome Age Index (TAI) across developmental stages. A) Global TAI computed using all analyzed genes. The profile displays peak evolutionary conservation (lowest TAI) during mid-embryogenesis (10.3–24 hpf), consistent with the developmental hourglass model. **B)** Cumulative TAI analysis. **C)** TAI calculated independently for each defined gene class.

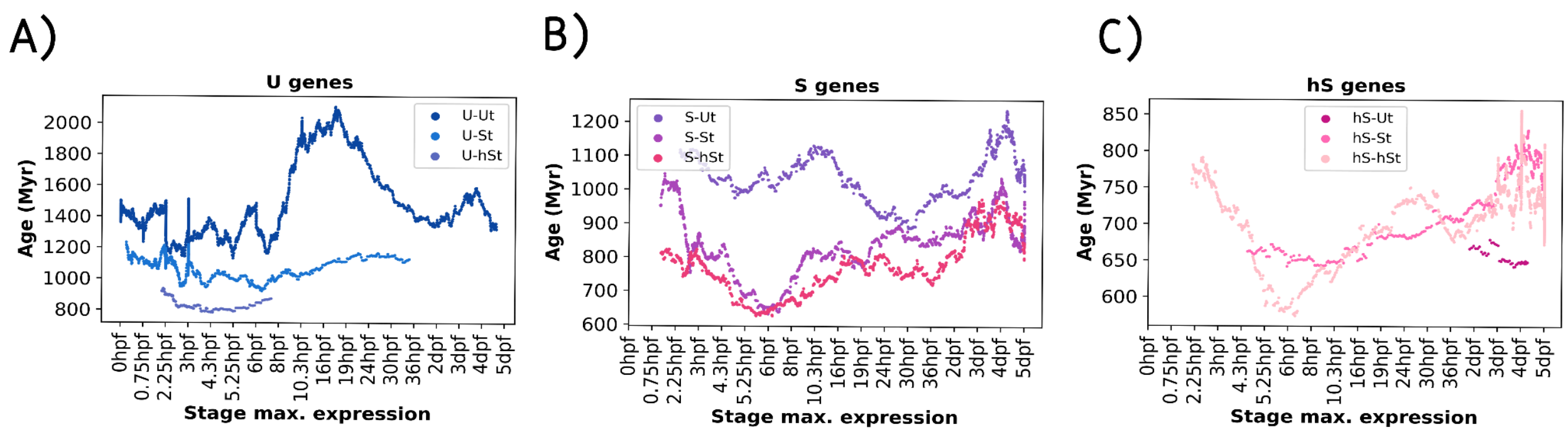

Fig SE. Evolutionary age and developmental timing of maximal gene expression across developmental categories and their adult tissue specificity. A) U genes. **B)** S genes. **C)** hS genes. Sliding window=200.

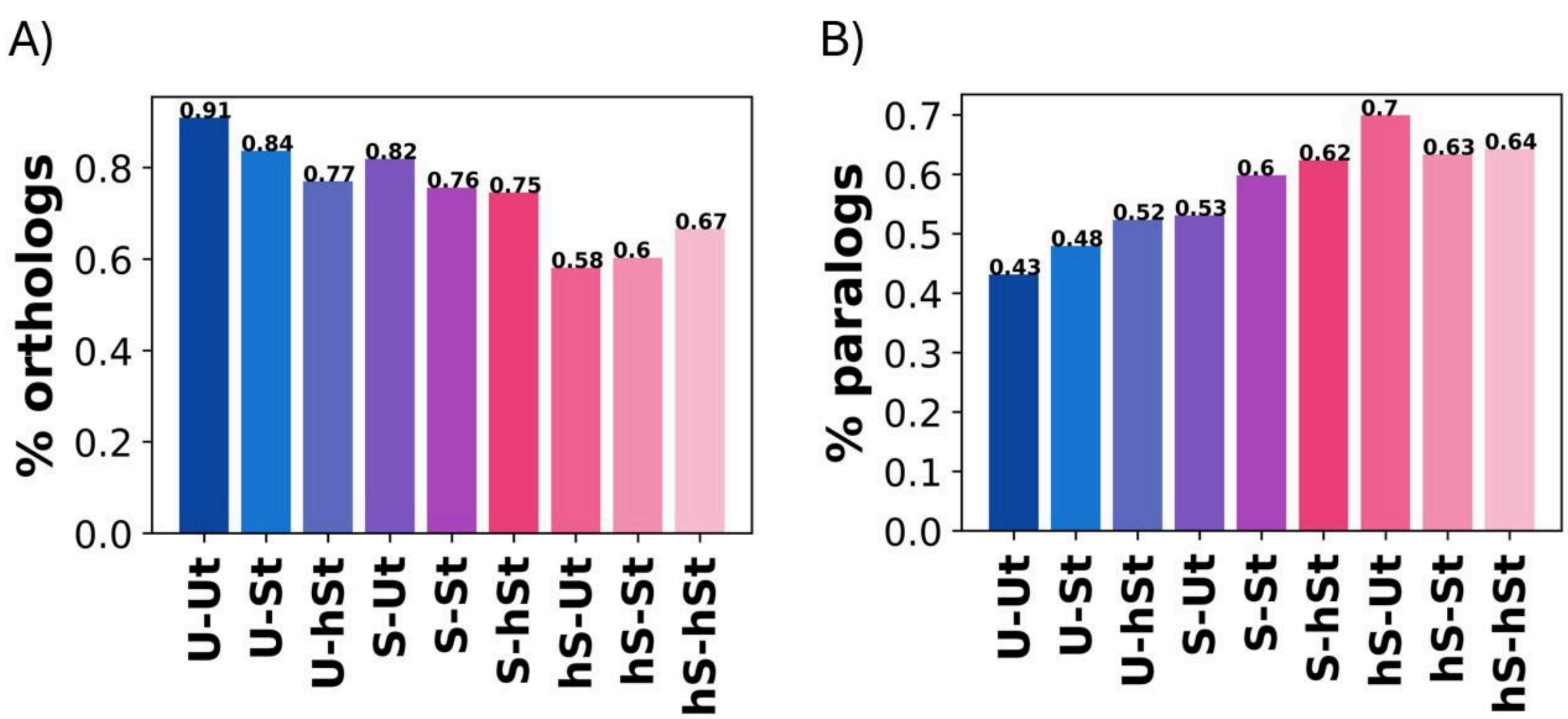

Fig SF. Analysis of gene homology per gene subclass. A) Fraction of genes within each subclass with at least one human ortholog. **B)** Fraction of genes within each subclass with at least one paralog. Older evolutionary genes (U-Ut; the oldest) tend to have more orthologs and fewer paralogs. In contrast, younger genes (hS-Ut; the youngest) usually have more paralogs (possibly due to gene duplications that support new functions of lineage-specific innovation) and fewer orthologs in humans, reflecting their lower evolutionary conservation and lineage specificity.

Analysis with continuous temporal specificity

To validate our findings, we extended our analysis using a continuous metric of temporal specificity (temporal τ). While our gene classifications stratify temporal and spatial specificity, here we take the complementary approach by directly analyzing temporal τ. We show that this continuous metric recapitulates similar patterns in GO enrichment, as well as in associations with tissue specificity and gene age.

Analysis of this continuous variable (Table S7) demonstrates that temporal specificity strongly aligns with distinct biological processes, successfully recapitulating the functional enrichment results of our discrete classification. Genes with high temporal specificity share the enriched Gene Ontology (GO) terms characteristic of the highly specific (hS) class (Table S2), which are primarily related to nervous system processes—specifically GPCR-driven cell signaling, ion transport, and synaptic transmission localized to neuronal structures. Conversely, genes with low temporal specificity mirror the ubiquitous (U) gene class (Table S2), showing strong enrichment for core cellular machinery, including gene expression, cellular metabolism, intracellular transport, and the mitotic cell cycle.

We next compared the continuous temporal specificity during development (temporal τ) with the continuous spatial specificity in adult tissues (spatial τ). When analyzing all genes collectively, we observed a significant positive correlation between the two expression specificities (Pearson's $r=0.67$; p-value<0.001). Broadly, expression restriction patterns established during development significantly overlap with those in adult tissues.

Although temporal and spatial τ are positively correlated (Fig SGA in S1 Text), comparing them revealed 848 outlier genes deviating heavily from the diagonal ($y=x$). Crucially, 87.6% of these continuous outliers correspond directly to our discrete classification deviations—genes that shift expression categories between development and adult tissue. To validate this statistically, we analyzed the residuals (Fig SGB in S1 Text) for all 16,953 mapped genes ($\mu=0.09$, $\sigma=0.20$). While most genes (70.2%) fell within 1σ, we identified 864 severe outliers (5.1%) located beyond 2σ. Strikingly, 85.6% of these extreme $>2\sigma$ outliers correspond directly to our discrete classification deviations. These severe outliers are primarily driven by: hS-Ut (20.1%), S-hSt (18.4%), hS-St (16.7%) and U-hSt (12.0%) genes.

Finally, we assessed the relationship between temporal specificity and evolutionary gene age (Fig SGC in S1 Text). We observed a significant negative correlation between the two variables across 21,238 common genes (Pearson's $r=-0.2723$; p-value<0.001). Consistent with our sliding window analysis, this anticorrelation demonstrates that phylogenetically younger genes tend to exhibit higher temporal specificity (aligning with the hS class profile), whereas more evolutionarily conserved genes exhibit lower temporal specificity (aligning with the U class profile).

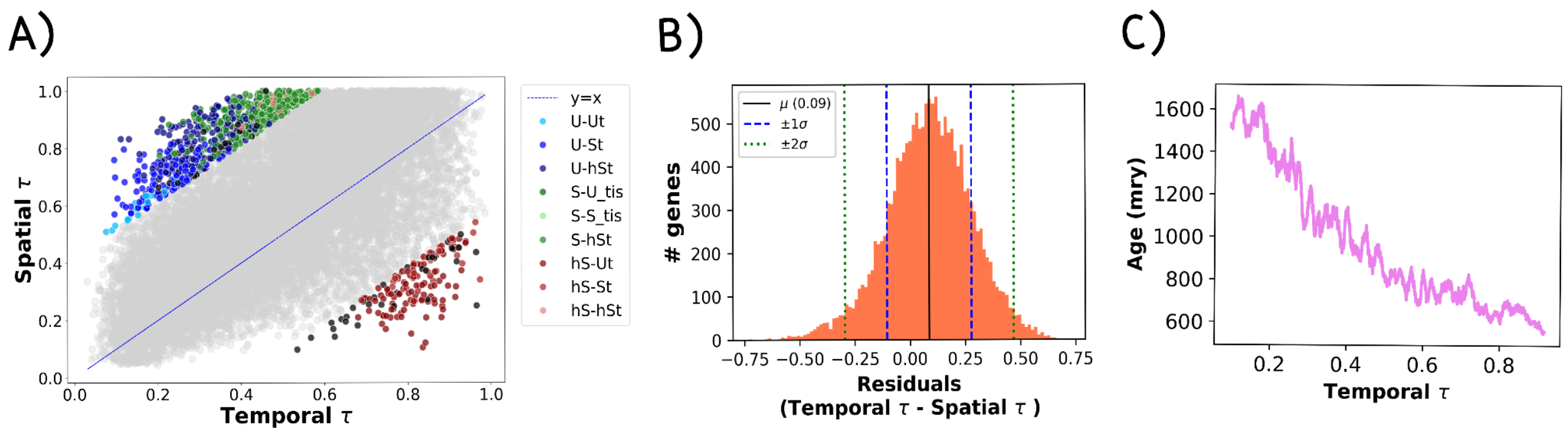

Fig SG. Analysis of continuous temporal specificity. A) Correlation between continuous temporal specificity and spatial specificity. Gray dots represent genes that follow the general correlated trend. Colored dots highlight the top 5% of outliers deviating most from the diagonal (y=x), color-coded by their discrete gene classification. The majority of these extreme outliers correspond to genes that shift classification categories between development and adult tissue. **B)** Distribution of the residuals from the diagonal in panel A. The central black line indicates the mean, blue lines represent one standard deviation (1σ), and green lines denote two standard deviations (2σ). **C)** Relationship between evolutionary gene age and temporal specificity. A sliding window analysis (sw=400) reveals a negative correlation: genes with high temporal specificity tend to be evolutionarily younger, whereas genes with low temporal specificity are generally older and more conserved.

Robustness to expression thresholds

To confirm that our biological conclusions are not artifacts of the chosen expression threshold (TPM > 1.0), we re-analyzed our core findings under a lower threshold (TPM > 0.5) and a higher threshold (TPM > 2.0).

We evaluated the stability of class membership across developmental stages and adult tissues. Overall, the classifications were highly stable. In the developmental dataset, parameter sweeps showed that most genes (>75%) retained their original ubiquitous (U), specific (S), or highly specific (hS) labels, indicating that the core identity of these gene sets is preserved even under extreme threshold changes (Fig SHA in S1 Text). A similarly robust core was observed in adult tissues, where at least 60–70% of genes consistently retained their original assignments (Fig SHB in S1 Text). The membership changes observed during parameter sweeps were biologically consistent. When a more permissive expression threshold (TPM > 0.5) was used, retention of hS and S genes decreased because low basal expression became detectable in more stages or tissues. As a result, some hS genes were reclassified as S, and some S genes as U. In contrast, a stricter threshold (TPM > 2.0) limited the number of stages or tissues in which genes were considered active, increasing apparent specificity and shifting genes from U to S and from S to hS.

Also, we confirmed that the evolutionary gene origin patterns remain completely intact regardless of the threshold. Both the distribution of gene ages across the U, S, and hS classes

(Fig SHC in S1 Text), as well as the sliding-window relationship between gene origin and the maximal stage of expression (Fig SHD in S1 Text), are perfectly conserved at TPM > 0.5, TPM > 1.0, and TPM > 2.0.

Finally we analyzed the stability of functional enrichment. Gene Ontology (GO) enrichments are highly robust to thresholding (Table SA in S1 Text). Across Biological Process and Molecular Function ontologies, between 72.6% and 95.7% of the significantly enriched terms discovered at TPM > 1.0 remained significant at both alternative thresholds across all classes. The only notable exception to the robust GO term preservation occurred within the Cellular Component ontology for the intermediate S class at the stringent TPM > 2.0 threshold. While U and hS Cellular Component terms retained >85% overlap, the S class dropped to a 37.5% retention rate (losing 10 out of 16 original terms).

The 10 terms lost under the TPM > 2.0 threshold are: extracellular matrix, small GTPase-mediated signal transduction, extracellular matrix structural constituent, anchoring junction, intermediate filament, intermediate filament cytoskeleton, extracellular region, external encapsulating structure, collagen-containing extracellular matrix and extracellular space.

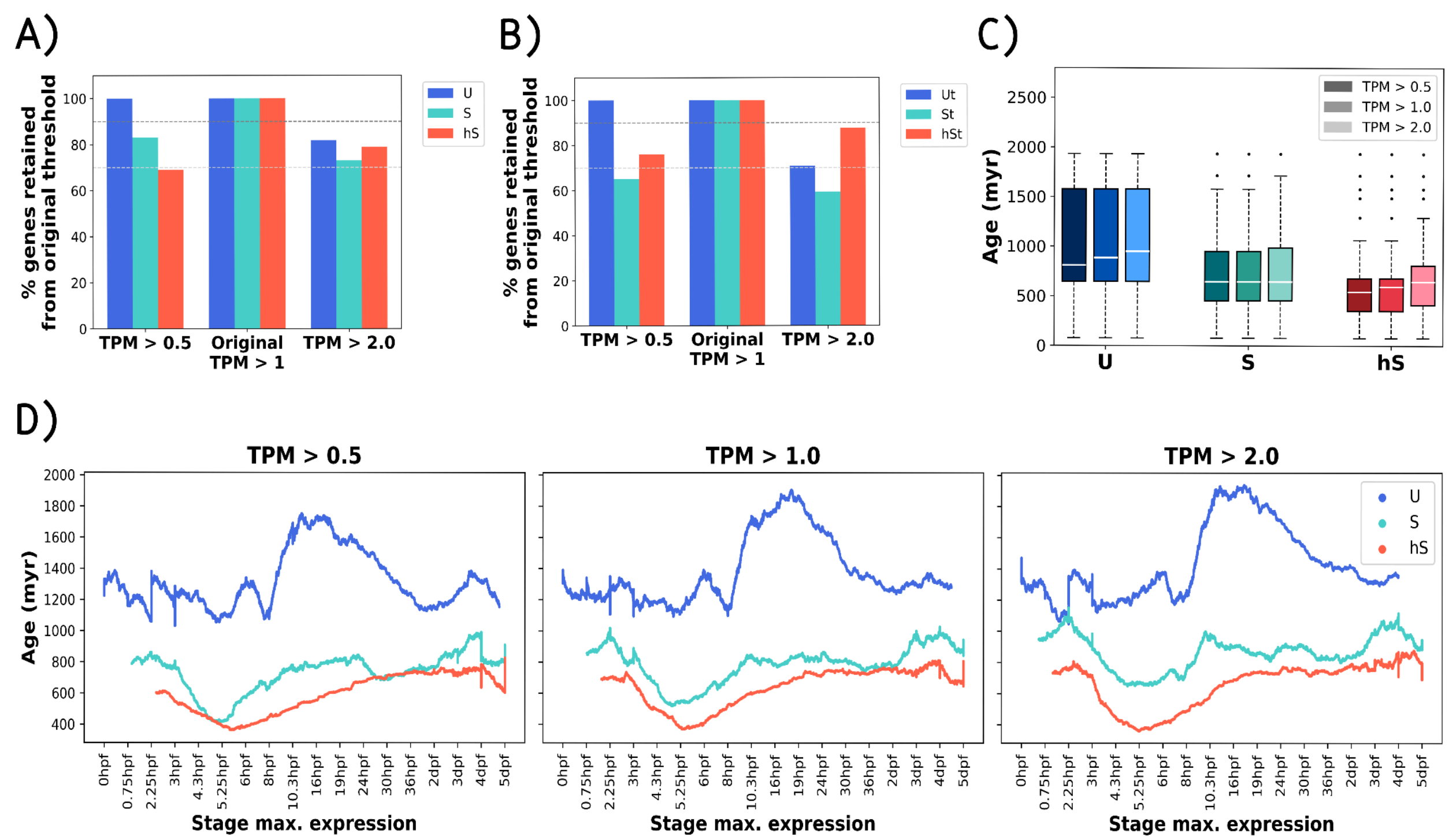

Fig SH. Robustness of gene classification and evolutionary trends to varying expression thresholds. A) Percentage of genes retained in their original developmental classes (U, S, hS) when altering the TPM expression cutoffs. **B)** Percentage of genes retained in their original tissue classes (Ut, St, hSt) when altering the TPM expression cutoffs. **C)** Evolutionary age distributions across gene classes under varying expression thresholds (in gene developmental classes), with color intensity indicating the stringency of the TPM cutoff. **D)** Relationship

between evolutionary gene age and the maximal stage of expression across different thresholds. Note: The TPM > 1.0 profile is identical to the main text analysis for reference.

**Ontology**	**Gene class**	Original significant terms **(TPM > 1.0)**	Retained at **TPM > 0.5**	Retained at **TPM > 2.0**
**Biological Process**	**U**	253	90.9%	95.7%
	**S**	156	79.5%	83.3%
	**hS**	197	89.8%	72.6%
**Molecular Function**	**U**	93	81.7%	87.1%
	**S**	25	80.0%	80.0%
	**hS**	124	89.5%	73.4%
**Cellular Component**	**U**	155	85.2%	97.4%
	**S**	16	87.5%	37.5%
	**hS**	85	91.8%	87.1%

Table SA. Stability of gene ontology (GO) enrichments across expression thresholds. The table displays the total number of significant GO terms identified under the original threshold (TPM > 1.0) and the percentage of those specific terms that remain statistically significant (p-value<0.0001) when applying relaxed (TPM > 0.5) or stringent (TPM > 2.0) expression cutoffs.

Validation of ubiquitous (U) gene expression via scRNA-seq

To verify that our identified ubiquitous (U) genes reflect true broad expression rather than bulk RNA-seq compositional artifacts (e.g., lineage-specific expansion or shared resident cell types across organs), we analyzed their distribution at single-cell resolution. Using developmental scRNA-seq data (Methods; (Lange et al., 2024), we mapped 8,355 developmental U genes (97.3% overlap) across 154 annotated cell types (Fig SIA in S1 Text). A gene was scored as "expressed" in a specific cell type if it was detected in that cell type during at least one developmental stage. Our analysis revealed that 99% of the mapped U genes were expressed in over 79% of the evaluated cell types. Similarly, using 22-month adult scRNA-seq data from the Zebrafish Cell Landscape (Methods; Wang et al., 2023), we mapped 5,745 adult U genes

(66% overlap) across 41 cell types, observing that 90% were expressed in more than 80% of the adult cell types (Fig SIB in S1 Text). Together, these analyses confirm that our identified U genes mostly maintain genuinely broad expression across diverse cell lineages and are not artifacts of shifting cell type composition.

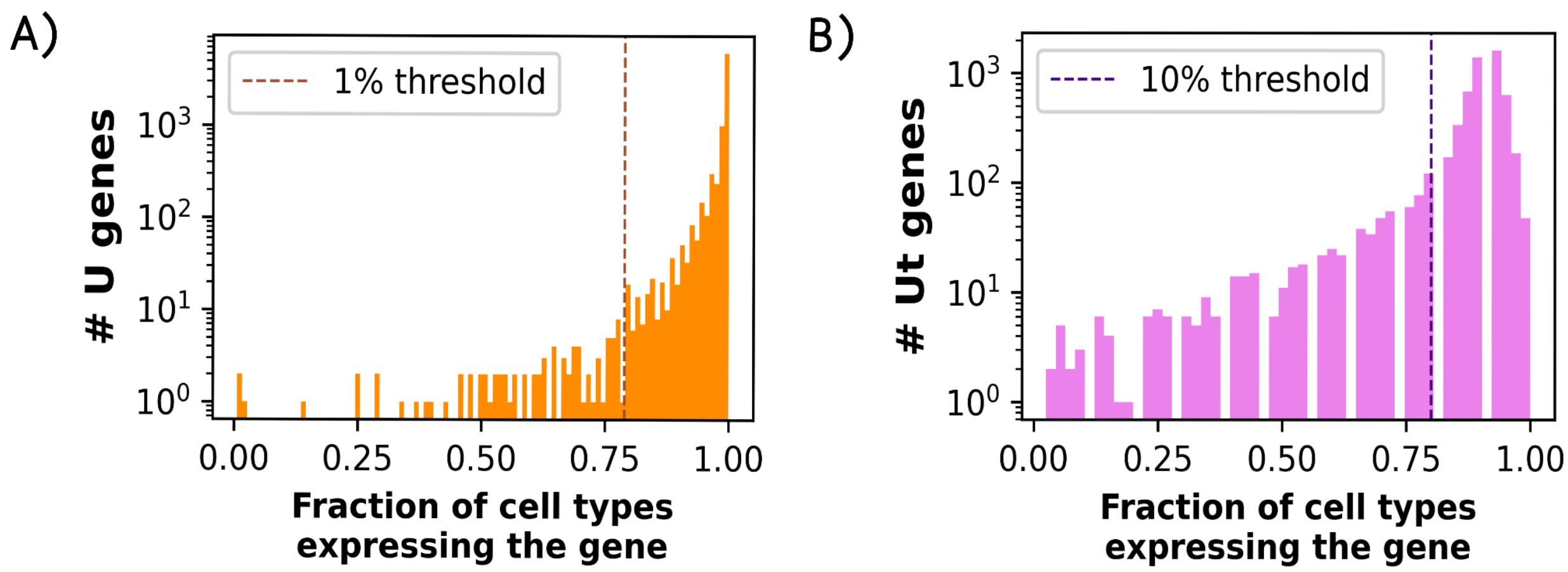

Fig SI. Single-cell expression breadth of ubiquitous (U) genes. Distribution of the fraction of annotated cell types expressing U genes in scRNA-seq datasets. **A)** Evaluation of U genes across 154 cell types (Lange et al., 2024). **B)** Evaluation of Ut genes across 41 cell types (Zebrafish Cell Landscape; Wang et al., 2023). In panel A, the vertical dashed line indicates the 1st percentile of the distribution, while in panel B, it indicates the 10th percentile. These thresholds mark the points above which 99% and 90% of the U genes are expressed, respectively. This corresponds to expression in >79% of developmental cell types and >80% of adult cell types, demonstrating their widespread expression across lineages.

References

Amsterdam, A., Nissen, R. M., Sun, Z., Swindell, E. C., Farrington, S., & Hopkins, N. (2004). Identification of 315 genes essential for early zebrafish development. *Proceedings of the National Academy of Sciences*, *101*(35), 12792–12797. https://doi.org/10.1073/pnas.0403929101

Bo, J., Fang, W., Wang, J., He, S., & Yang, L. (2025). High resolution of full-length RNA sequencing deciphers massive transcriptome complexity during zebrafish embryogenesis. *BMC Biology*, *23*(1), 155. https://doi.org/10.1186/s12915-025-02271-2

Domazet-Lošo, T., & Tautz, D. (2010). A phylogenetically based transcriptome age index mirrors ontogenetic divergence patterns. *Nature*, *468*(7325), 815–818. https://doi.org/10.1038/nature09632

Hu, P., Liu, M., Zhang, D., Wang, J., Niu, H., Liu, Y., Wu, Z., Han, B., Zhai, W., Shen, Y., & Chen, L. (2015). Global identification of the genetic networks and *cis* -regulatory elements of the cold response in zebrafish. *Nucleic Acids Research*, *43*(19), 9198–9213. https://doi.org/10.1093/nar/gkv780

Lange, M., Granados, A., VijayKumar, S., Bragantini, J., Ancheta, S., Kim, Y.-J., Santhosh, S., Borja, M., Kobayashi, H., McGeever, E., Solak, A. C., Yang, B., Zhao, X., Liu, Y., Detweiler, A. M., Paul, S., Theodoro, I., Mekonen, H., Charlton, C., … Royer, L. A. (2024). A multimodal zebrafish developmental atlas reveals the state-transition dynamics of late-vertebrate pluripotent axial progenitors. *Cell*, *187*(23), 6742-6759.e17. https://doi.org/10.1016/j.cell.2024.09.047

Lou, A., Chagoyen, M., & Poyatos, J. F. (2026). Cell atlases and the developmental foundations of the phenotype. *PLOS Computational Biology*, *22*(2), e1013944. https://doi.org/10.1371/journal.pcbi.1013944

Stoeger, T., Gerlach, M., Morimoto, R. I., & Nunes Amaral, L. A. (2018). Large-scale investigation of the reasons why potentially important genes are ignored. *PLOS Biology*, *16*(9), e2006643. https://doi.org/10.1371/journal.pbio.2006643

Wang, R., Zhang, P., Wang, J., Ma, L., E, W., Suo, S., Jiang, M., Li, J., Chen, H., Sun, H., Fei, L., Zhou, Z., Zhou, Y., Chen, Y., Zhang, W., Wang, X., Mei, Y., Sun, Z., Yu, C., … Han, X. (2023). Construction of a cross-species cell landscape at single-cell level. *Nucleic Acids Research*, *51*(2), 501–516. https://doi.org/10.1093/nar/gkac633

White, R. J., Collins, J. E., Sealy, I. M., Wali, N., Dooley, C. M., Digby, Z., Stemple, D. L., Murphy, D. N., Billis, K., Hourlier, T., Füllgrabe, A., Davis, M. P., Enright, A. J., & Busch-Nentwich, E. M. (2017). A high-resolution mRNA expression time course of embryonic development in zebrafish. *eLife*, *6*, e30860. https://doi.org/10.7554/eLife.30860

Yanai, I., Benjamin, H., Shmoish, M., Chalifa-Caspi, V., Shklar, M., Ophir, R., Bar-Even, A., Horn-Saban, S., Safran, M., Domany, E., Lancet, D., & Shmueli, O. (2005). Genome-wide midrange transcription profiles reveal expression level relationships in human tissue specification. *Bioinformatics*, *21*(5), 650–659. https://doi.org/10.1093/bioinformatics/bti042
